# Fine-tuning acetyl-CoA carboxylase 1 activity through localization: functional genomics reveals a role for the lysine acetyltransferase NuA4 and sphingolipid metabolism in regulating Acc1 activity and localization

**DOI:** 10.1093/genetics/iyac086

**Published:** 2022-05-24

**Authors:** Trang Pham, Elizabeth Walden, Sylvain Huard, John Pezacki, Morgan D Fullerton, Kristin Baetz

**Affiliations:** Ottawa Institute of Systems Biology, University of Ottawa, Ottawa, ON K1H 8M5, Canada; Department of Biochemistry, Microbiology and Immunology, Faculty of Medicine, University of Ottawa, Ottawa, ON K1H 8M5, Canada; Ottawa Institute of Systems Biology, University of Ottawa, Ottawa, ON K1H 8M5, Canada; Department of Biochemistry, Microbiology and Immunology, Faculty of Medicine, University of Ottawa, Ottawa, ON K1H 8M5, Canada; Ottawa Institute of Systems Biology, University of Ottawa, Ottawa, ON K1H 8M5, Canada; Department of Biochemistry, Microbiology and Immunology, Faculty of Medicine, University of Ottawa, Ottawa, ON K1H 8M5, Canada; Ottawa Institute of Systems Biology, University of Ottawa, Ottawa, ON K1H 8M5, Canada; Department of Chemistry and Biomolecular Sciences, Faculty of Science, University of Ottawa, Ottawa, ON K1N6N5, Canada; Department of Biochemistry, Microbiology and Immunology, Faculty of Medicine, University of Ottawa, Ottawa, ON K1H 8M5, Canada; Ottawa Institute of Systems Biology, University of Ottawa, Ottawa, ON K1H 8M5, Canada; Department of Biochemistry, Microbiology and Immunology, Faculty of Medicine, University of Ottawa, Ottawa, ON K1H 8M5, Canada; Department of Biological Sciences, Faculty of Science, University of Calgary, Calgary, AB T2N 1N4, Canada

**Keywords:** NuA4, lysine acetylation, Acc1, protein localization, protein aggregation, fatty acids, *Saccharomyces cerevisiae*

## Abstract

Acetyl-CoA Carboxylase 1 catalyzes the conversion of acetyl-CoA to malonyl-CoA, the committed step of *de novo* fatty acid synthesis. As a master regulator of lipid synthesis, acetyl-CoA carboxylase 1 has been proposed to be a therapeutic target for numerous metabolic diseases. We have shown that acetyl-CoA carboxylase 1 activity is reduced in the absence of the lysine acetyltransferase NuA4 in *Saccharomyces cerevisiae*. This change in acetyl-CoA carboxylase 1 activity is correlated with a change in localization. In wild-type cells, acetyl-CoA carboxylase 1 is localized throughout the cytoplasm in small punctate and rod-like structures. However, in NuA4 mutants, acetyl-CoA carboxylase 1 localization becomes diffuse. To uncover mechanisms regulating acetyl-CoA carboxylase 1 localization, we performed a microscopy screen to identify other deletion mutants that impact acetyl-CoA carboxylase 1 localization and then measured acetyl-CoA carboxylase 1 activity in these mutants through chemical genetics and biochemical assays. Three phenotypes were identified. Mutants with hyper-active acetyl-CoA carboxylase 1 form 1 or 2 rod-like structures centrally within the cytoplasm, mutants with mid-low acetyl-CoA carboxylase 1 activity displayed diffuse acetyl-CoA carboxylase 1, while the mutants with the lowest acetyl-CoA carboxylase 1 activity (hypomorphs) formed thick rod-like acetyl-CoA carboxylase 1 structures at the periphery of the cell. All the acetyl-CoA carboxylase 1 hypomorphic mutants were implicated in sphingolipid metabolism or very long-chain fatty acid elongation and in common, their deletion causes an accumulation of palmitoyl-CoA. Through exogenous lipid treatments, enzyme inhibitors, and genetics, we determined that increasing palmitoyl-CoA levels inhibits acetyl-CoA carboxylase 1 activity and remodels acetyl-CoA carboxylase 1 localization. Together this study suggests yeast cells have developed a dynamic feed-back mechanism in which downstream products of acetyl-CoA carboxylase 1 can fine-tune the rate of fatty acid synthesis.

## Introduction

Fatty acids (FAs) are a key building block of life and while they are taken up from human diet, they can also be synthesized *de novo* in the cell (Mashima *et al.* 2009). In the highly proliferative conditions of cancer, changes in cellular energetics are a hallmark, and *de novo* lipogenesis provides lipids for membrane formation allowing for proliferation (Mashima *et al.* 2009; Hanahan and Weinberg 2011). In fact, regulation and therapeutic targeting of FA availability have been proposed to limit cancer cell growth (Mashima *et al.* 2009; Currie *et al.* 2013). As a key pathway in lipid metabolism, de novo FA synthesis is also relevant to obesity and nonalcoholic fatty liver disease (Fullerton *et al.* 2013; Engin 2017; Rios Garcia *et al.* 2017). Acetyl-CoA carboxylase (ACC) converts acetyl-CoA into malonyl-CoA creating the committed precursor for FA synthetase to produce long-chain FAs (Wakil and Abu-Elheiga 2009). The process is conserved across many forms of life ranging from humans to fungi including *Saccharomyces cerevisiae* (Witters and Watts 1990; Tehlivets *et al.* 2007; Nielsen 2009). In *S. cerevisiae*, there are 2 *ACC* proteins: Acc1, which functions in the cytoplasm and Hfa1, which functions in the mitochondria (Tehlivets *et al.* 2007; Kim *et al.* 2016).

As the enzyme catalyzing the initial and committed step of de novo lipid synthesis (lipogenesis), Acc1 activity is highly regulated by multiple mechanisms (Witters and Watts 1990; Brownsey *et al.* 2006). First, Acc1 is regulated at the transcriptional level through the Ino2/Ino4 transcriptional activator complex and the Opi1 repressor (Chirala *et al.* 1994) and *ACC1* mRNA is upregulated when phosphatidic acid (PA) levels are high (Carman and Han 2011). In addition, Acc1 protein levels are also regulated at the translational level, peaking at G2/M during the cell cycle which is controlled by an upstream open reading frame (uORF) and plays a role in response to nutrient availability, as mutation of the uORF increased expression of Acc1 in a poor carbon source, 3% glycerol (Blank *et al.* 2017). This is not driven by changes in the abundance of *ACC1* mRNA but by increases in translation of Acc1 protein (Blank *et al.* 2017). In both mammals and yeast, Acc1 activity is tightly regulated by phosphorylation, largely by the AMP-activated protein kinase, AMPK, or Snf1 in yeast (Witters and Watts 1990; Woods *et al.* 1994; Winder *et al.* 1997; Fullerton *et al.* 2013). When Snf1/AMPK activity is induced upon low-glucose or starvation conditions, Snf1/AMPK phosphorylation of Acc1 inhibits its activity, resulting in a decrease in FA synthesis (Witters and Watts 1990; Woods *et al.* 1994). In yeast, the primary Snf1 phosphorylation site on Acc1 is serine 1157 and under logarithmic growth over 60% of Acc1 protein is phosphorylated at this site suggesting the majority of enzyme is inactive under this condition (Hofbauer *et al.* 2014). In *snf1Δ* cells and in *acc1^S1157A^* mutant cells, where the Snf1-S1157 phosphorylation site is mutated to mimic the unphosphorylated state, Acc1 activity is hyperactive, resulting in high levels of FA production, accumulation of TAG, and lipid droplets during logarithmic growth (Hofbauer *et al.* 2014). As the first step of FA synthesis, Acc1 also influences FA chain length. In mutants with low Acc1 activity (hypomorphs) or upon inhibition of Acc1, acyl-chain length decreases whereas in mutants with high Acc1 activity (hypermorphs), such as *acc1^S1157A^*^,^ acyl-chain length increases (Schneiter *et al.* 1996, 2000; Hofbauer *et al.* 2014).

In addition, many other pathways influence Acc1 activity. In human cells, citrate allosterically activates ACC activity (Witters and Watts 1990; Tehlivets *et al.* 2007). Citrate regulation is also associated with a polymerization of human Acc1 into long filaments where the Acc1 is locked into the active form (Hunkeler *et al.* 2018). Remarkably, downstream products of Acc1 can also negatively impact Acc1 activity forming a negative feedback loop. In mammalian cells, exogenous palmitoyl-CoA reduces Acc1 activity through an alteration of the Acc1 conformation and disruption of filament structure (Hunkeler *et al.* 2018). Exogenous long-chain FAs inhibit Acc1 activity in yeast extracts (Kamiryo *et al.* 1976; Shirra *et al.* 2001) and 16–20 carbon long-chain length acyl-CoAs directly inhibit rat liver Acc1 (Ogiwara *et al.* 1978; Nikawa *et al.* 1979), though the exact mechanism of inhibition is not known. In addition, very long-chain FA (VLCFA)-CoAs can feedback onto Acc1 activity through allosteric activation of AMPK (Pinkosky *et al.* 2020). Acc1 is also inhibited by Soraphen A (Sor A), a compound discovered from *Sonrangium cellulosum*, a myxobacteria, which was initially used as an antifungal but has branched into similar compounds being used in clinical trials (Gerth *et al.* 1994; Vahlensieck *et al.* 1994; Naini *et al.* 2019). Sor A is commonly used as an indirect measurement of Acc1 activity as mutants deficient for Acc1 activity are hypersensitive and mutants with enhanced Acc1 activity (such as Snf1 or *acc1^S1157A^* mutants) are resistant to Sor A treatment (Shirra *et al.* 2001; Bozaquel-Morais *et al.* 2017; Dacquay *et al.* 2017).

Our lab has recently determined that in mutants of the yeast lysine acetyltransferase (KAT) NuA4 complex, Acc1 activity is decreased and intracellular levels of acetyl-CoA are increased (Rollins *et al.* 2017). The NuA4 complex is composed of 13 subunits, including the essential catalytic subunit Esa1 (Clarke *et al.* 1999). The cellular activity of NuA4 can be modulated through use of the temperature-sensitive allele of *ESA1*, *esa1-L254P* (*esa1-ts*; Clarke *et al.* 1999). NuA4 activity can also be reduced through the deletion of *EAF1*, a key scaffolding protein of the complex, and deletion of another member, *EAF7*, demonstrates phenotypes associated with NuA4 impairment (Krogan *et al.* 2004; Mitchell *et al.* 2008, 2011; Rollins *et al.* 2017). Though NuA4 was named for its acetylation of Histone H4, it has a growing list of cellular roles and nonhistone targets (Lin *et al.* 2008, 2009; Lu *et al.* 2011; Huang *et al.* 2018). In fact, roles for NuA4 have been identified for multiple aspects of lipid homeostasis. NuA4 has been implicated in the negative regulation of Snf1 (AMPK) through the acetylation of Sip2, one of the 3 Snf1 inhibitory subunits (Lu *et al.* 2011). This acetylation increases the interaction of Sip2 with the Snf1 complex, thereby reducing Snf1 activity (Lu *et al.* 2011). Another target of NuA4 acetylation is Kes1/Osh4, a yeast oxysterol binding protein (Huang *et al.* 2018). Acetylation at the K109 site decreases Kes1 lipid exchange activity and a reduction in NuA4 activity led to an enhancement in Kes1 activity (Huang *et al.* 2018). NuA4 has also been implicated in phospholipid homeostasis through the acetylation of PA phosphatase, Pah1, the enzyme which catalyzes the PA to diacylglycerol step of glycerolipid synthesis (Han *et al.* 2006; Li *et al.* 2018). Finally, NuA4 mutants display decreases in lipid droplet formation (Dacquay *et al.* 2017; Li *et al.* 2018).

Given the central role of Acc1 in metabolism and disease and the increasing use of KAT inhibitors to treat various diseases (Brown *et al.* 2016; Di Martile *et al.* 2016; Fiorentino *et al.* 2020), we were motivated to uncover the mechanisms by which NuA4 regulates Acc1 activity. Despite the established role of NuA4 in regulating Snf1/AMPK, we determined that NuA4 regulation of Acc1 activity is only partially through Snf1/AMPK. Surprisingly, we found that NuA4 influences the subcellular localization of Acc1, which impacts its enzymatic activity. Extending this discovery, we performed a targeted genetic screen for changes in Acc1-GFP localization and identified additional mutants that impacted Acc1 localization and activity, which suggests that in yeast, Acc1 localization and activity are linked. Finally, our data implicate NuA4 in the regulation of sphingolipid flux and palmitoyl-CoA levels which in turn can fine-tune Acc1 activity.

## Materials and methods

### Yeast strains and growth conditions

*Saccharomyces* *cerevisiae* strains used in this study are listed in Supplementary Table 2. Strains were generated by standard mating procedure or PCR-based genetic deletion/epitope tagging as previously described (Longtine *et al.* 1998). Point mutation S1157A of Acc1 was introduced into wild-type (wt) strain that expressed GFP-tagged Acc1 at its genomic locus by CRISPR-Cas9 method as previously described (DiCarlo *et al.* 2013). All strains were confirmed by genomic PCR or sequencing with specific primers. Deletion strains for Acc1 localization screen were from the Deletion Mutant Array collection (GE, catalog no. YSC1053). Yeast were grown in standard YPD medium (1% yeast extract, 2% peptone, 2% dextrose), synthetic complete (SC) or drop-out medium (2% dextrose, 0.67% yeast nitrogen base without amino acids, and 0.2% amino acid complete or drop-out mix) at 30°C unless otherwise specified.

Cells were imaged, subjected to specific treatments, or harvested for further investigation in early logarithmic (log) phase (OD_600_ of 0.4–0.5). For treatment with cerulenin (Sigma, Cat#C2389), myristic acid (Sigma, Cat#M3128), palmitic acid (Sigma, Cat#P0500), stearic acid (Sigma, Cat#S4751), ceramide (Tocris, Cat#0744), Sor A (Collaboration with John Pezacki and Rolf Muller; Wenzel *et al.* 2006), and myriocin (Sigma, Cat#M1177), log phase cells were centrifuged at 3,000 rpm for 3 min at room temperature and resuspended in media to which the appropriate drug was added to desired concentration immediately prior to use. Brij58 detergent (Sigma, Cat#P5884) was included in the media at a concentration of 0.1% to solubilize cerulenin, FAs, and ceramide.

### Yeast microscopy and image quantification

Yeast strains that express GFP tagged Acc1 were grown in YPD or SC media overnight, diluted to an OD_600_ of 0.1, and cultured at 30°C to early-log phase (OD_600_ of 0.4–0.5). Alternatively, early-log phase cells were treated with different compound(s) as indicated. For imaging, 50 μl of the culture was added to a well on a SensoPlate 96-well black microplate with glass bottom (Greiner Bio-one, Cat# 82050-792) that was pretreated with concanavalin A (Sigma, Cat# L7647). The plate was then centrifuged at 500 rpm at room temperature for 1 min. Cells adhered to the bottom of the well were washed once with SC media or SC media containing appropriate compound(s) and then resuspended in the same media. A z-stack of 30 fluorescent and brightfield images across 12 μm were captured with CellVoyager CV1000 disk confocal imaging system equipped with back-illuminated EMCCD camera and 100× objective lens (Olympus Life Science) at room temperature. At least 3 independent biological replicates for each yeast strain and condition were performed with at least 100 cells/replicate. Cells and Acc1 structures were identified and quantified by Imaris ×64 9.1.0 software (Bitplane) using 2 separate algorithms for automatic segmentation. Both algorithms used the green fluorescent channel that depicts Acc1-GFP localization since Acc1 is localized to both the distinct structures and the cytoplasm. To identify and quantify cells, a surface was modeled based on the average size of a yeast cell with a smoothing factor of twice the pixel size and a lower intensity threshold. Smoothing was omitted and the intensity threshold was increased to model the Acc1 structures. The statistical parameters of interest included the number of disconnected objects and intensity sum (for both cells and Acc1 structures), the intensity average, and average volume (for Acc1 structures).

### Cell lysis and Acc1 purification

Cells harvested from 100 ml culture of OD_600_ 0.4–0.5 were lysed in 1 ml lysis buffer [mChIP buffer (100 mM HEPES pH8.0, 20 mM magnesium acetate, 200 mM sodium acetate, 10 mM EGTA, 0.1 mM EDTA, 10% glycerol) supplemented with mini protease inhibitor cocktail tablet (Roche Cat# 4693159001), 50 mM sodium butyrate (Sigma, Cat#B5887), 50 mM nicotinamide (Sigma, Cat#72340), 5 μM TSA (Sigma, Cat#EPI008), 0.4 μM apicidin (Sigma, Cat#EPI008), 2 μM M344 (Sigma, Cat#EPI008), 1 mM sodium valproate (Sigma, Cat#EPI008), 1 mM sodium orthovanadate (Sigma, Cat#S6506), 50 mM sodium fluoride (Sigma, Cat#201154), and phosphatase cocktail 2 and 3 (Sigma, Cat#P5726 and P0044, respectively)]. To this mix, 500 μl glass beads (Fisher Scientific, USA; 35–535) was added and bead beating was performed for a total of ten 1-min periods with 1-min interval on ice. Crude cell lysates were centrifuged at 1,000 rpm for 1 min at 4°C. Protein concentration of the cloudy whole-cell extract was determined with Bio-Rad Protein Assay Dye Reagent (Bio-Rad, Cat# 5000006). When indicated, 1% NP40 was included in the lysis buffer to solubilize Acc1 and in that case crude cell lysate was centrifuged at 13,200 rpm for 15 min at 4°C to obtain a clear extract.

For purification of GFP-tagged Acc1, 50 μl of GFP-trap magnetic beads (ChromoTek, Cat#GTM-100) were incubated with the cell extract by rotating end-over-end for 2 h at 4°C. If cell extract contains NP40, the beads were washed 3 times with 1 ml mChIP buffer with 1% NP40 followed by a wash with 1 ml mChIP buffer without NP40. Otherwise, if cell extract does not contain NP40, it is omitted from the wash buffer. Washed beads were resuspended in 50 μl mChIP buffer without NP40 and used immediately for Acc1 assay. Equal amounts of sample were also run on a 6% SDS-PAGE gel followed by coomassie staining with simpleblue safestain coomassie (Thermo Fisher Scientific, Cat#LC6060) for normalization of Acc1 activity.

Tandem affinity purification of TAP tagged Acc1 was performed by incubation of the cell extract with 200 μl of IgG magnetic beads prepared as described previously (Mitchell *et al.* 2013) for 2 h at 4°C. The beads were washed as described earlier prior to an additional wash with 1 ml elution buffer (50 mM Tris pH 8.0, 1 mM EDTA, 1 mM DTT, 150 mM NaCl, and 10% Glycerol). Acc1 was released from the beads by cleavage with 10 U of ScTEV (Thermo Fisher Scientific, Cat#12575015) in 50 μl of elution buffer overnight at 4°C and was used immediately for Acc1 assay and SDS-PAGE as described above.

### Acc1 activity assay

The Acc1 assay was performed as previously described (Rollins *et al.* 2017). Briefly, equal volumes of bead slurry that contains purified Acc1-GFP were incubated with reaction buffer [50 mM HEPES pH 7.4, 10 mM MgCl_2_, 1 mM MnCl_2_, 2 mM DTT, 0.4 mM ATP, 0.075% FA free BSA (Sigma, cat # A7030), 12.5 mM NaHCO3 and 1.5 µCi 14C-NaHCO_3_ (Perkin Elmer, cat# NEC086H005MC)] by rotating end-over-end. Palmitoyl-CoA was included in the reaction at 10 µM when indicated. The reactions were incubated at room temperature for 90 min, stopped by the addition of 0.6 N HCl, and air-dried overnight at 37°C. The radioactivity of the products was determined by scintillation counting of the dried materials. Acc1 activity was normalized to relative protein abundance in the samples as determined by coomassie staining of SDS-PAGE gels. Protein quantification was performed with Image Lab5.2 software.

### Yeast growth assay

Yeast growth was quantitatively monitored by the Bioscreen C automated microbiology growth curve analysis system (Growth Curve USA) that measures the optical density at 600 nm (OD_600_) of microcultures on a honeycomb 100-well plate (Growth Curve USA, 9502550). Log phase cells (note: BY4741 background without Acc1 tagging) were seeded in 200 μl YPD media that contain appropriate treatment. The plate was incubated at 30°C with constant shaking at high amplitude and speed and OD_600_ was measured every 15 min for 2 days. The raw OD_600_ readings were processed by Pregcog software (Fernandez-Ricaud *et al.* 2016) to obtain adjusted OD_600_ values that estimate population size. These values were used to generate the growth curves and calculate the growth rates. Growth rate was defined as the slope of the best-fit line when the natural logarithm of the adjusted OD was graphed vs time between 1 h and the first time point when the adjusted OD_600_ reaches 25% of the maximum OD_600_. Three biological replicates, each of which has 2–3 technical replicates, were performed. The growth rates of the technical replicates were averaged and these average values of the 3 biological replicates were used for statistical analysis as indicated.

### Lipogenesis assay

Lipogenesis assay was performed by pulse labeling early-log phase WT and mutant cells grown in YPD medium with 1 μCi/ml ^3^H-sodium acetate for 1 h at 30°C. For drug treatment, log phase cells were centrifuged at 3000 rpm at room temperature for 3 min and resuspended in YPD medium containing 0.1% Brij58 and appropriate drugs. Cells were then cultured for 2 h at 30°C before pulse labeling with ^3^H-sodium acetate as described above. An equal number of cells were collected for harvest for each sample for both protein and lipid extractions. Lipids were extracted using Bligh and Dyer method (Bligh and Dyer 1959) with some modifications. Radioactively labeled cells were lysed in 300 μl of methanol:chloroform (2:1) and 80-μl glass beads (Fisher Scientific, USA; 35–535) by vortexing for a total of six 1-min periods with 1-min interval on ice. Lipid was extracted by the sequential addition of 100 μl chloroform and 100 μl water. Following centrifugation at 5,000 ×g for 4 min at 4°C, the bottom lipid-containing chloroform layer was recovered and subjected to scintillation counting. Lipogenesis was determined as the radioactivity of the extracted lipid normalized to the protein concentration of lysate made from the same number of cells used for lipid extraction. Protein lysate was made by bead beating cells with 100 μl mChIP buffer (100 mM Hepes pH8.0, 20 mM magnesium acetate, 200 mM sodium acetate, 10 mM EGTA, 0.1 mM EDTA, 10% glycerol) supplemented with protease inhibitor cocktail (Sigma; P8215) and 80 μl glass beads. Protein concentrations of the clarified lysates were measured by microBCA kit (Thermo Scientific Cat#23232) or Bio-Rad protein assay dye reagent (Bio-Rad, Cat# 5000006).

### SILAC labeling, purification, and liquid chromatography-mass spectrometry/MS analysis of Acc1-TAP

Yeast SILAC labeling was performed as previously described (Downey *et al.* 2015) with light (K0) or heavy (K6) lysine (Cambridge Isotope Laboratories, Cat#ULM-8766-0.1/CLM-2247-H-0.1, respectively). The heavy lysine has a mass shift of +6 Da compared to the light one. Wild-type and *esa1-ts* strains that express TAP tagged Acc1 in a *lys2Δ* background were grown at 30°C to near saturation in synthetic lysine drop-out medium (SC-Lys) supplemented with 30 mg/l of either K0 or K6 overnight before being diluted in the same labeling medium and cultured to near saturation again for a second day. Then cells were diluted to an OD_600_ of 0.1 in 200 ml of the same labeling medium and grown at 30°C to an OD_600_ of 0.2–0.25. Yeast culture was shifted to 34°C for 2 h when its OD_600_ reached 0.45–0.55. Cells were harvested by centrifugation at 3,000 rpm for 5 min at 4°C. Cells were washed with 2 ml cold water and snap-frozen. Cell lysis and TAP purification of Acc1 were performed using lysis buffer that contained 1% NP40 as described earlier except that after incubation of lysate with IgG beads, beads from the light and heavy samples were combined prior to 4 wash steps with 1 ml of mChIP buffer that contains 1% NP40. Acc1-TAP was eluted from the beads by heating at 65°C in 40 μl of 2% SDS for 10 min twice. Acc1 was reduced by boiling of the combined SDS eluant in 10 mM DTT (Sigma, Cat#D9779) at 100°C for 10 min. Cysteine alkylation was achieved by incubating the reduced samples with fresh 50 mM 2-iodoacetamide (Sigma, Cat#I1149) at room temperature in the dark for 30 min. NP LDS sample buffer (Thermo Fisher Scientific, Cat#NP0007) was added to the sample prior to protein separation on a 3–8% Tris-Acetate NuPAGE gel (Thermo Fisher Scientific, Cat#EA0375BOX) and coomassie staining with simpleblue safestain coomassie (Thermo Fisher Scientific, Cat#LC6060) according to the manufacture instructions. In-gel digestion and peptide recovery were performed as previously described (Trinkle-Mulcahy 2012). Acc1 band was excised from the gel, minced into small pieces with a clean sharp scalpel. All washing and extraction steps were done on a shaking platform at room temperature unless otherwise indicated. Gel pieces were washed with 300 μl of liquid chromatography-mass spectrometry (LC-MS) grade water (Thermo Fisher Scientific, Cat# W61) for 15 min. To this 300 μl of LC-MS grade acetonitrile (Thermo Fisher Scientific, Cat#A9551) was added, and samples were shaken for another 15 min. After removal of the liquid, gel pieces were sequentially washed with 300 μl of 20 mM ammonium biocarbonate (Thermo Fisher Scientific, Cat#A643) and 300 μl of 20 mM Ammonium biocarbonate: acetonitrile (50:50 v/v) for 15 min each. Gel pieces were dehydrated by 100 μl acetonitrile for 5 min and dried in a speedvac. Trypsin digestion was performed with 630 ng of LC-MS grade trypsin (Thermo Fisher Scientific, Cat#PI90058) in 20 mM ammonium biocarbonate at 37°C overnight. Peptide was eluted by addition of an equal volume of acetonitrile and incubation at 30°C for 30 min followed by 2 extractions with 100 μl of 1% LC-MC grade formic acid (Thermo Fisher Scientific, Cat#A11750) and one with 200 μl of acetonitrile. All peptide extracts were combined and completely dried in a speedvac. Peptides were cleaned up using Pierce C18 spin tips (Thermo Fisher Scientific, Cat#PI84850) and then dried in a speedvac.

Proteomics analysis was performed at the Ottawa Hospital Research Institute Proteomics Core Facility (Ottawa, Canada). LC-MS/MS was performed using a Dionex Ultimate 3000 RLSC nano HPLC (Thermo Scientific) and Orbitrap Fusion Lumos mass spectrometer (Thermo Scientific). Peptide identification and quantification were performed by MaxQuant 1.5.5.1 software using *S. cerevisiae* sequence database from SwissProt (version 2016-03).

### Mini screen for Acc1 localization

The microscopic screen to identify genetic deletions that affect Acc1 localization was performed by combining synthetic genetic array analysis (SGA) and high-throughput imaging according to a method previously described (Chong *et al.* 2015; Styles *et al.* 2016) with some modifications. An array of 84 Acc1-GFP expressing deletion mutants was created by crossing a mat α Acc1-GFP expressing query strain (*ura3*Δ::natMX *his3*Δ*1 leu*Δ*0 ura3*Δ*0 MET15 can1pr::RPL39pr-tdTomato::CaURA3 can1* Δ*::STE2pr-LEU2 ACC1-GFP-HIS3*) and a set of corresponding mutants obtained from the yeast haploid deletion collection (Supplementary Table 1) using SGA method. The deletion mutants chosen were involved in processes that might regulate Acc1 according to the literature and our own observations (i.e. sphingolipid, citrate, glucose metabolism, glucose sensor, and signaling pathways).

SGA procedure was performed by ROTOR HDA (Singer Instruments). After sporulation of the diploids resulted from mating of the query strain and the selected gene deletion array, mating type a haploid deletion mutants were selected for by pinning cells on synthetic medium that lacks leucine and arginine but contains 50 µg/ml canavanine, 200 µg/ml G418, and 100 µg/ml nourseothricin (NAT). Subsequent selections were performed 3 times on synthetic medium that lacks leucine, arginine, and histidine but contains canavanine, G418, and NAT to select for strains with both gene deletions and Acc1-GFP expression.

The resultant Acc1-GFP expressing deletion mutants were cultured overnight in complete synthetic medium at 25°C in 96 deep well plates. If the OD_600_ of overnight cultures were above 0.6, the cultures were diluted to an OD_600_ of 0.1, and grown at 25°C until OD_600_ reached 0.4–0.6 (early-mid log) when cells were imaged. Otherwise, cells were imaged when OD_600_ of overnight culture was 0.4–0.6. Image acquisition and analysis were performed as described in the microscopy section above. Strains that showed abnormal Acc1-GFP localization as compared to that in wt strain based on 2 independent imaging experiments were repeated twice more with growing at 30°C instead of 25°C.

### FAS activity assay

FAS activity was determined in cell extracts at 25°C as previously described (Lynen 1969; Matias *et al.* 2011). Yeast (note: BY4741 background without Acc1 tagging) were grown to early-log phase (OD_600_ of 0.4–0.5) in YPD medium and 50 OD_600_ of cells was harvested. Cells were lysed in 600 μl lysis buffer [100 mM potassium phosphate buffer pH 7.5 supplemented with mini protease inhibitor cocktail (Roche Cat# 4693159001), 1 mM PMSF, 50 mM sodium butyrate (Sigma, Cat#B5887), 50 mM nicotinamide (Sigma, Cat#72340), 5 μM TSA (Sigma, Cat#EPI008), 0.4 μM apicidin (Sigma, Cat#EPI008), 2 μM M344 (Sigma, Cat#EPI008), 1 mM sodium orthovanadate (Sigma, Cat#S6506), 50 mM sodium fluoride (Sigma, Cat#201154), and phosphatase cocktail 2 and 3 (Sigma, Cat#P5726 and P0044, respectively)] and 500 μl glass beads (Fisher Scientific, USA; 35–535) by bead beating method. Crude cell lysates were centrifuged at 8,000 ×g for 20 min at 4°C. One hundred microliters of clarified extracts were added to 900 μl of FAS assay buffer [100 mM potassium phosphate buffer pH 6.5, 2.5 mM EDTA, 10 mM cysteine, 0.3 mg/ml FA free BSA (Sigma, Cat#A7030), 0.06 mM acetyl-CoA (Sigma, Cat#A2056), and 0.15 mM NADPH (Sigma, Cat#N9960)] in a 1 ml cuvette. The blank rate of NADPH oxidation (Ro) was recorded as the rate of decrease in absorbance at 340 nm for 3 min using Ultrospec 2100 Pro spectrophotometer. The reaction was then started by the addition of 10 μl of 7 mM malonyl-CoA (final concentration of 0.07 mM; Sigma, Cat#M4263) and the total rate of NADPH oxidation (Rx) was recorded as described above. The rate of reaction was calculated by subtracting the blank rate from the total rate (Rx-Ro). One milliunit (mU) of enzyme is defined as the amount of enzyme which consumes 1 nmol of malonyl-CoA per minute (corresponding to 2 nmol of NADPH or a change of 0.006 in absorbance at 340 nm) under this reaction condition. Specific activity is expressed as milliunit per milligram of protein (mU/mg). Protein concentration of cell extracts was performed using microBCA kit (Thermo Scientific Cat#23232) according to the manufacture instruction.

### Lipidomic analysis

To prepare samples for lipidomic analysis, overnight culture of wt and *eaf1Δ* cells (note: BY4741 background without Acc1 tagging) were diluted to an OD_600_ of 0.1 in YPD medium and grown at 30°C to early-log phase (OD_600_ of 0.4–0.5) before being harvested. For Sor A treatment, log phase wt cells were diluted 100 times in YPD medium that contains 0.3 μM SorA and grown for 24 h at 30°C. Twenty OD_600_ of yeast cells were harvested by centrifugation at 4,000 ×g for 3 min at 4°C. Cells were washed once with 10 ml and once with 1 ml of cold milliQ water. Cell pellets were snap-frozen and stored at −80°C. Cells were lysed in 1 ml LC-MS grade water and 500-μl glass beads using a bead beater for a total of ten 1-min beating periods with 1-min chilling interval. Five hundred microliters of crude lysate was used for lipid extraction.

Mass spectrometry-based lipid analysis was performed by Lipotype GmbH (Dresden, Germany) as described previously (Ejsing *et al.* 2009; Klose *et al.* 2012). Lipids were extracted using a 2-step chloroform/methanol procedure (Ejsing *et al.* 2009). Samples were spiked with internal lipid standard mixture containing: CDP-DAG 17:0/18:1, ceramide 18:1; 2/17:0 (Cer), diacylglycerol 17:0/17:0 (DAG), lyso-phosphatidate 17:0 (LPA), lyso-phosphatidylcholine 12:0 (LPC), lyso-phosphatidylethanolamine 17:1 (LPE), lyso-phosphatidylinositol 17:1 (LPI), lyso-phosphatidylserine 17:1 (LPS), phosphatidate 17:0/14:1 (PA), phosphatidylcholine 17:0/14:1 (PC), phosphatidylethanolamine 17:0/14:1 (PE), phosphatidylglycerol 17:0/14:1 (PG), phosphatidylinositol 17:0/14:1 (PI), phosphatidylserine 17:0/14:1 (PS), ergosterol ester 13:0 (EE), triacylglycerol 17:0/17:0/17:0 (TAG), inositolphosphorylceramide 44:0; 2 (IPC), mannosyl-inositolphosphorylceramide 44:0; 2 (MIPC), and mannosyl-di-(inositolphosphoryl)ceramide 44:0; 2 [M(IP)2C]. After extraction, the organic phase was transferred to an infusion plate and dried in a speed vacuum concentrator. First step dry extract was resuspended in 7.5 mM ammonium acetate in chloroform/methanol/propanol (1:2:4, V:V:V) and second step dry extract in 33% ethanol solution of methylamine in chloroform/methanol (0.003:5:1; V:V:V). All liquid handling steps were performed using Hamilton Robotics STARlet robotic platform with the Anti Droplet Control feature for organic solvents pipetting.

To acquire MS data, samples were analyzed by direct infusion on a QExactive mass spectrometer (Thermo Scientific) equipped with a TriVersa NanoMate ion source (Advion Biosciences). Samples were analyzed in both positive and negative ion modes with a resolution of Rm/z = 200 = 280,000 for MS and Rm/z = 200 = 17,500 for MSMS experiments, in a single acquisition. MSMS was triggered by an inclusion list encompassing corresponding MS mass ranges scanned in 1 Da increments (Surma *et al.* 2015). Both MS and MSMS data were combined to monitor EE, DAG, and TAG ions as ammonium adducts; PC as an acetate adduct; and CL, PA, PE, PG, PI, and PS as deprotonated anions. MS only was used to monitor LPA, LPE, LPI, LPS, IPC, MIPC, M(IP)2C as deprotonated anions; Cer and LPC as acetate adducts.

Data were analyzed with in-house developed lipid identification software based on LipidXplorer (Herzog *et al.* 2011, 2012). Data postprocessing and normalization were performed using an in-house developed data management system. Only lipid identifications with a signal-to-noise ratio >5, and a signal intensity 5 times above the corresponding blank samples were considered for further data analysis. For diacylglycerol and phospholipid classes whose subspecies were quantified (i.e. DAG, PA, PI, PC, PS, PE, and PG), quantities of different individual FAs were derived from the quantities of the lipid species. This was then used to determine the percent of total lipids for each class. Additionally, the number if carbon atoms in the hydrocarbon for each lipid class were estimated by calculating the average length of the chain per lipid class. While data were collected for all the above listed lipids, in this paper, we show a subset containing the most highly identified species. All Lipotype data are available in Supplementary Table 3.

### Statistical analysis

Statistical analysis was performed with GraphPad Prism 9 software as indicated in the figure legends.

## Results

### Reduction of Acc1 activity in *eaf1*Δ cells is independent of SNF1/AMPK1

**Fig. 1.**
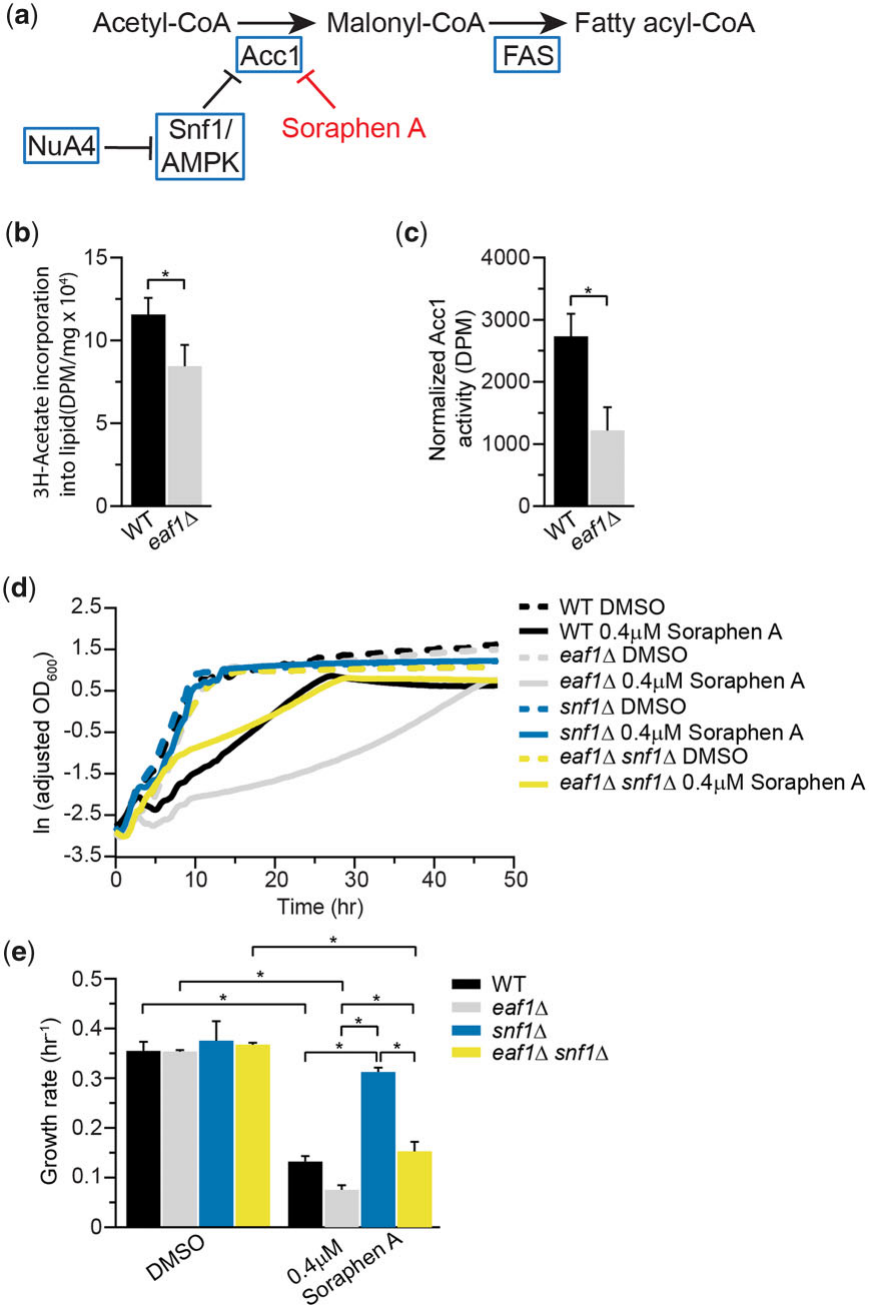
NuA4-dependent regulation of Acc1 activity is not solely through Snf1/AMPK1. a) Schematic of de novo synthesis pathway of FAs. The precursor of FA synthesis is acetyl-CoA. The essential enzyme Acc1 plays a direct role in regulating FA synthesis. Genetic manipulations of yeast cells (NuA4 mutants) or pharmacological inhibition (Sor A) impacting Acc1 activity can attenuate the synthesis of FAs. b) Deletion of the NuA4 subunit, *EAF1*, reduces the rate of triglyceride synthesis. Early-log phase wt (YKB 1079) and *eaf1Δ* (YKB 3333) cells were treated with [^3^H]-sodium acetate for 1 h before harvest and extraction. The lipogenesis rate was obtained by normalizing the incorporation of [^3^H] into triglycerides from [^3^H]-acetate precursor to the protein concentration obtained from the same cells. Error bars denote the standard error of the mean (SEM). *n* = 3, *P < 0.05 determined using a 2-tailed *t*-test. c) Acc1 activity is reduced in NuA4 mutant. Wild-type (YKB 3954) and *eaf1Δ* (YKB 4448) cells expressing endogenously tagged Acc1-GFP were grown to early-log phase at 30°C in YPD. Acc1-GFP was immunoprecipitated and Acc1 activity measured and normalized to Acc1-GFP protein abundance. Error bars denote the standard error of the mean (SEM). *n* = 3, *P < 0.05 determined using a 2-tailed *t*-test. d) *eaf1Δ* cells are sensitive to Sor A and *eaf1Δ snf1Δ* cells are more sensitive than *snf1Δ* cells. Wild-type (YKB 1079), *eaf1Δ* (YKB 3333), *snf1Δ* (YKB 3389), and *eaf1Δ snf1Δ* (YKB 3421) cells were grown to early-log phase before being diluted to an OD_600_ of 0.1 in YPD with DMSO control or 0.4 µM Sor A in DMSO, and automated growth curve analysis was performed at 30°C for 48 h. e) Growth rates of indicated strains were calculated from logarithmic growth phase of curves in d. Error bars denote the standard error of the mean (SEM). *n* = 3, *P < 0.05 determined using a 2-way ANOVA test with a Tukey’s multiple comparisons test.

We had previously shown that Acc1 activity, but not protein levels, are reduced in whole-cell extracts from cells in which *EAF1* is deleted (Rollins *et al.* 2017). To confirm that the impact of deletion of *EAF1* on Acc1 activity is not an artifact of whole-cell extracts, we performed a series of experiments. As Acc1 can serve as a limiting step of FA biosynthesis (Al-Feel *et al.* 1992; Hasslacher *et al.* 1993; Chirala *et al.* 1994; Woods *et al.* 1994), wt and *eaf1Δ* cells were treated with ^3^H-labelled acetate and its incorporation into lipids was measured. In agreement with reduced Acc1 activity, deletion of *EAF1* reduces lipogenesis rates (Fig. 1b). We next performed *in vitro* Acc1 activity assays using Acc1-GFP purified from wt and *eaf1*Δ cells and determined that the *in vitro* activity of Acc1-GFP purified from *eaf1*Δ cells was reduced (Fig. 1c). It is well established that mutants that have decreased Acc1 activity also display increased sensitivity to, or decreased growth rates, upon treatment with the highly specific Acc1 inhibitor Sor A (Gerth *et al.* 1994; Vahlensieck *et al.* 1994; Wei and Tong 2015). Therefore, we performed growth rate analysis comparing the impact of Sor A treatment on WT and *eaf1*Δ cells. Control and *eaf1Δ* cells had similar growth rates under untreated conditions (DMSO), but while Sor A treatment slightly reduced growth in the wt, the growth rate of *eaf1Δ* cells was further reduced, suggesting that *eaf1Δ* cells have less Acc1 activity than WT (Fig. 1, d and e). This is in agreement with a chemical genomic screen which identified the NuA4 subunit mutant *eaf7*Δ as hypersensitive to Sor A (Bozaquel-Morais *et al.* 2017). As previous studies have shown that NuA4 inhibits Snf1/AMPK activity (Lu *et al.* 2011), a key regulator of Acc1, we next sought to determine if the reduction in Acc1 activity in *eaf1*Δ cells was fully or partially due to increases in Snf1 activity. Therefore, we extended the Sor A growth rate studies to *snf1Δ* and *snf1Δ eaf1Δ* cells. If the decrease in Acc1 activity displayed by *eaf1*Δ cells was only due to increased Snf1 activity, one would predict the *snf1*Δ *eaf1*Δ cells would be resistant to Sor A treatment identical to that of *snf1*Δ cells. Instead, *snf1*Δ *eaf1*Δ cells displayed Sor A sensitivity similar to wt cells, suggesting that deletion of *SNF1* only partially suppresses the decrease of Acc1 activity in *eaf1Δ* cells. Said another way, the hyperactive Acc1 seen in *snf1*Δ cells can be repressed by deletion of *EAF1*, which suggests NuA4 must be modulating Acc1 activity through pathways independent of Snf1. Taken together, our work indicates that NuA4-dependent regulation of Acc1 activity is not solely through Snf1, but that parallel mechanisms occur.

### Acc1-GFP localization is modified by NuA4, Snf1, and phosphorylation state of Acc1

**Fig. 2.**
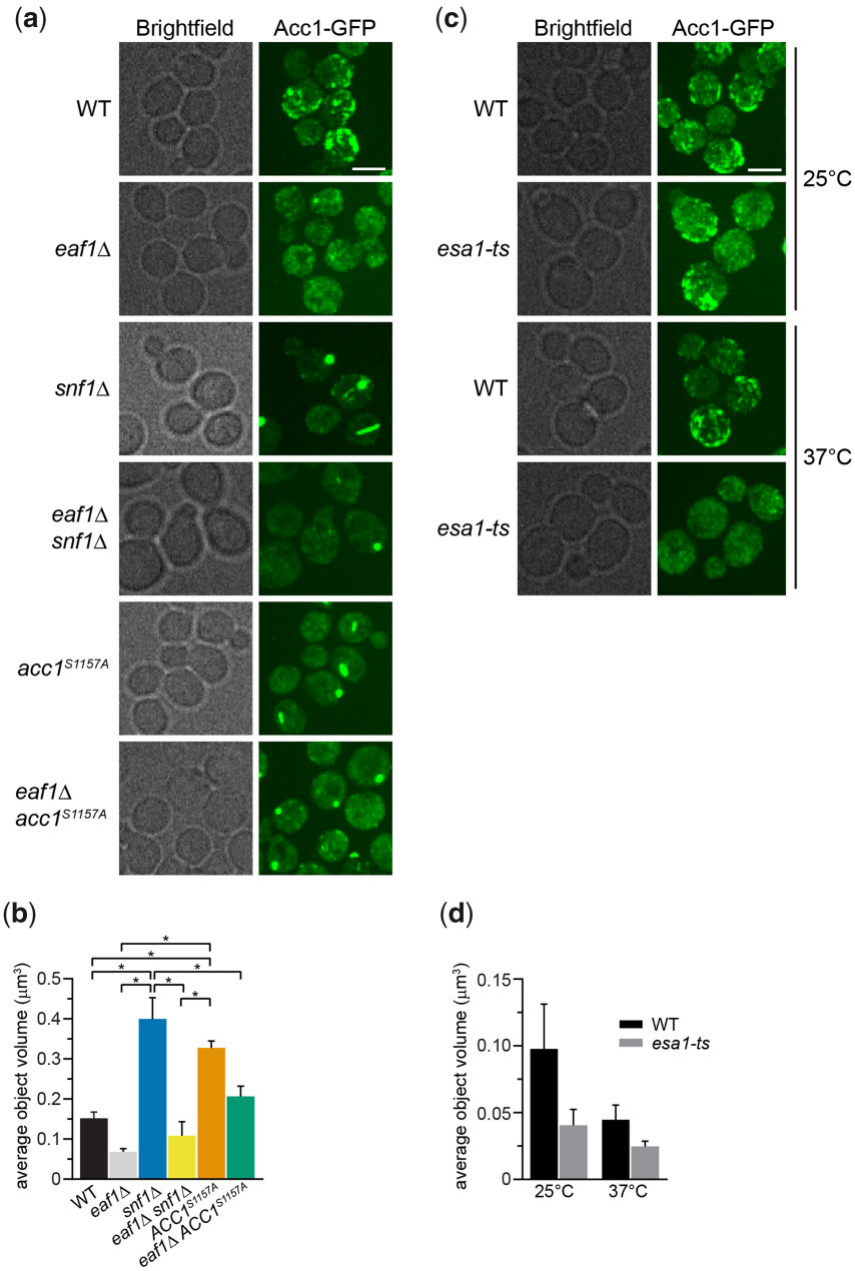
NuA4, Snf1, and Acc1 phosphorylation state mutants impact the localization of Acc1-GFP. a) Wild-type (YKB 3954), *eaf1Δ* (YKB 4448), *snf1Δ* (YKB 4348), *eaf1Δ snf1Δ* (YKB 4364), *acc1^S1157A^* (YKB 4401), and *eaf1Δ acc1^S1157A^* (YKB 4404) cells expressing endogenously tagged Acc1-GFP were grown to early-log phase at 30°C in YPD and assessed for Acc1-GFP localization. Representative brightfield and fluorescent images are shown. Scale bar: 4 µm. b) The average volume of Acc1-GFP structures in each strain was quantified using IMARIS software. Quantification is the average of 3 biological replicates, a minimum of 100 cells per replicate was scored. Error bars denote the standard error of the mean (SEM). *n* = 3, *P < 0.05 determined using a 1-way ANOVA test with a Tukey’s multiple comparisons test. c) Wild-type (YKB 3954) and *esa1-ts* (YKB 4303) yeast expressing endogenously tagged Acc1-GFP were grown to early-log phase at the permissive temperature of 25°C in YPD. Cells were then diluted to an OD_600_ of 0.2 in YPD and grown at restrictive temperature of 37°C for 2 h before Acc1-GFP localization was assessed. Representative brightfield and fluorescent images at 25°C and 37°C are shown. Scale bar: 4 µm. d) The average volume of Acc1-GFP structures in wt and *esa1-ts* strains at 25°C and 37°C were quantified using IMARIS software. Quantification is the average of 3 biological replicates, a minimum of 100 cells per replicate was scored. Error bars denote the standard error of the mean (SEM). *n* = 3, *P < 0.05 determined using a 2-way ANOVA test with a Tukey’s multiple comparisons test.

We next sought to discover the Snf1-independent pathways through which NuA4 impacts Acc1 activity. As numerous acetylome studies have detected acetylation sites on Acc1 (Henriksen *et al.* 2012; Weinert *et al.* 2014; Madsen *et al.* 2015), one possibility is that NuA4 is regulating the activity of Acc1 by direct acetylation. Therefore, we performed SILAC-based quantitative proteomics experiments to identify any Esa1-dependent (catalytic domain of NuA4) acetylation sites on purified Acc1-TAP (Supplementary Fig. 1). Though numerous acetylations were detected on Acc1, we did not detect any Esa1-dependent sites. We cannot exclude the possibility that there may be NuA4-dependent acetylation sites undetected by this method, but our work suggests that other mechanisms may be contributing to NuA4’s regulation of Acc1. As the subcellular localization of Acc1 has been reported to be dynamic in both directed (Wolinski *et al.* 2009) and high-throughput studies (Breker *et al.* 2013; Chong *et al.* 2015), we next asked if NuA4 impacted the localization of Acc1-GFP. Localization of Acc1 was assessed in wt and *eaf1Δ* cells expressing Acc1-GFP from its endogenous genomic location. As the localization of Acc1-GFP modulates depending on culture conditions (Wolinski *et al.* 2009), all localization studies were performed using rigorously controlled growth conditions, mid-log phase cells (OD_600_ 0.4–0.5) cultured at 30°C in complete media. Furthermore, to facilitate subsequent screens, images were obtained using a high-throughput CV1000 confocal microscope, which has poor brightfield imaging. In wt cells, Acc1-GFP was found in cytoplasmic punctate structures (Fig. 2a), similar to what has been previously reported (Breker *et al.* 2014; Chong *et al.* 2015). However, in *eaf1Δ* cells, the Acc1-GFP signal becomes diffuse throughout the cytoplasm with a significant decrease in the average punctate volume (object volume; Fig. 2b). We also assessed the impact of a temperature-sensitive allele of *ESA1*, *esa1-L254P* (*esa1-ts*) (Clarke *et al.* 1999), on Acc1-GFP localization at both a semipermissive temperature of 25°C and after 2 h at the nonpermissive temperature of 37°C (Fig. 2c). At both, the semipermissive temperature, where *esa1-ts* activity is partially reduced, and the restrictive temperature of 37°C, we detected a decrease in Acc1-GFP foci and a small but not significant decrease in average object volume (Fig. 2, c and d).

Our work indicated that the NuA4 complex regulates both the activity and the subcellular localization of Acc1, but we did not know if these were correlated. Therefore, we assessed Acc1-GFP localization in strains where Acc1 is hyperactive, *snf1*Δ and *acc1*^S1157A^ (Woods *et al.* 1994; Hofbauer *et al.* 2014). Remarkably, in both these strains, Acc1-GFP forms fewer foci, but the average punctate volumes are significantly larger (Fig. 2, a and b), and 3D reconstruction indicates these structures resemble cylinders or straight thick rods located in the center of the cell (Supplementary Fig. 2). Because of the straight cylinder shape, they appear on 2D images as either round foci or long rods. As *snf1*Δ partially rescues *eaf1*Δ sensitivity to Sor A (Fig. 1), we next assessed Acc1-GFP localization in both *snf1Δ eaf1Δ* and *eaf1Δ acc1*^S1157A^ backgrounds. In both cases, while distinct foci were still formed, they were smaller in average volume, and we detected an increase in overall cytoplasmic localization (Fig. 2, a and b). These results suggest that the localization of Acc1-GFP is associated with its activity, with large punctate dots forming in the hyperactive form, while the diffuse cytoplasmic pattern is correlated with decreased activity.

### FA elongation cycle and serine palmitoyltransferase contribute to Acc1 activity and localization

**Fig. 3.**
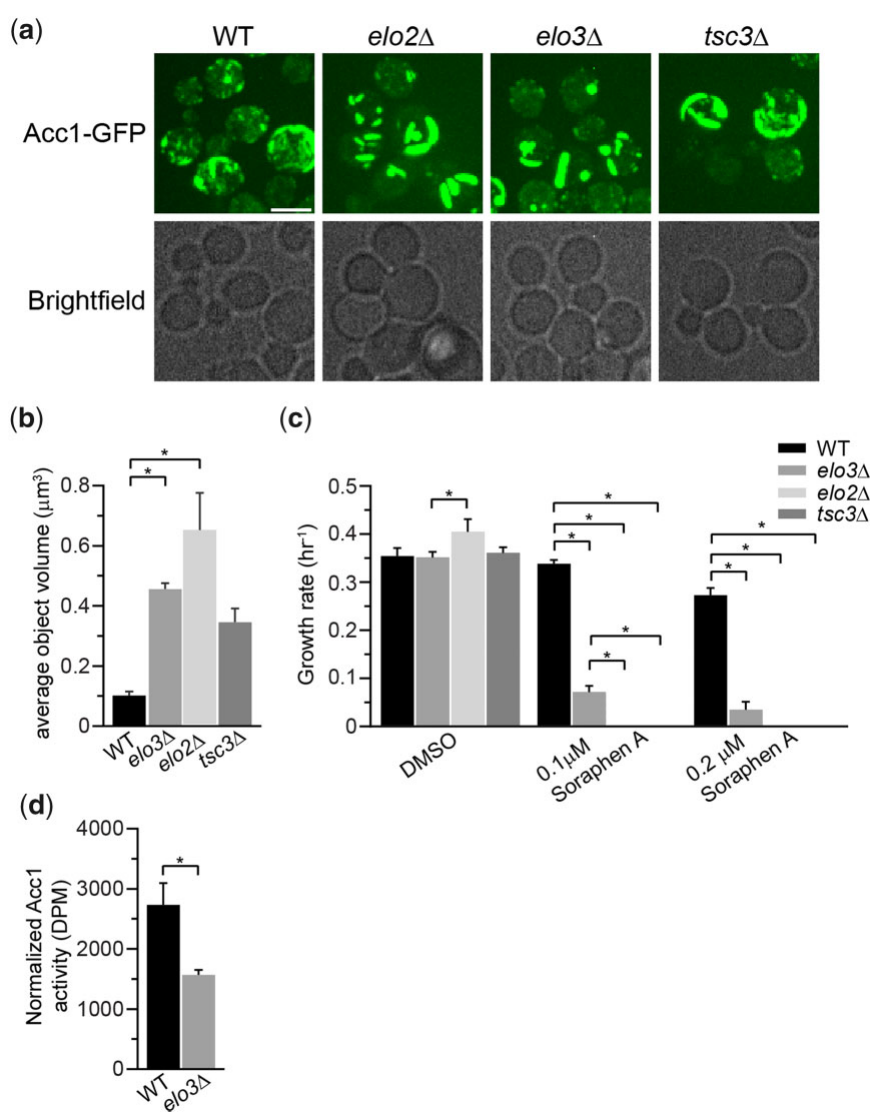
Genes from the FA elongation cycle and sphingolipid metabolism impact the localization and the activity of Acc1-GFP. a) Deletion of *ELO2*, *ELO3*, and *TSC3* impacts the localization of Acc1-GFP. Wild-type (YKB 3954), *elo2Δ* (YKB 4593), *elo3Δ* (YKB 4594), and *tsc3Δ* (YKB 4599) cells expressing endogenously tagged Acc1-GFP were grown to early-log phase at 30°C in YPD and assessed for Acc1-GFP localization. Representative brightfield and fluorescent images are shown. Scale bar: 4 µm. b) The average volume of Acc1-GFP structures in each strain was quantified using IMARIS software. Quantification is the average of 3 biological replicates, a minimum of 100 cells per replicate was scored. Error bars denote the standard error of the mean (SEM). *n* = 3, *P < 0.05 determined using a 1-way ANOVA test with a Tukey’s multiple comparisons test. c) *elo2Δ*, *elo3Δ*, and *tsc3Δ* cells display increased sensitivity to Sor A. Wild-type (YKB 1079), *elo2Δ* (YKB 3913), *elo3Δ* (YKB 3914), and *tsc3Δ* (YKB 3228) cells were grown to early-log phase before being diluted to an OD_600_ of 0.1 in YPD with DMSO control or 0.1 µM or 0.2 µM Sor A in DMSO, and an automated growth curve analysis was performed at 30°C for 48 h. Growth rate was calculated from 3 biological replicates. Error bars denote the standard error of the mean (SEM). *n* = 3, *P < 0.05 determined using a 2-way ANOVA test with a Tukey’s multiple comparisons test. d) Acc1 activity is reduced in *elo3Δ* cells. Wild-type (YKB 3954) and *elo3Δ* (YKB 4594) cells expressing endogenously tagged Acc1-GFP were grown to early-log phase at 30°C in YPD. Acc1-GFP was immunoprecipitated and Acc1 activity measured and normalized to Acc1-GFP protein abundance. Error bars denote the standard error of the mean (SEM). *n* = 3, *P < 0.05 determined using a 2-tailed *t*-test.

**Fig. 4.**
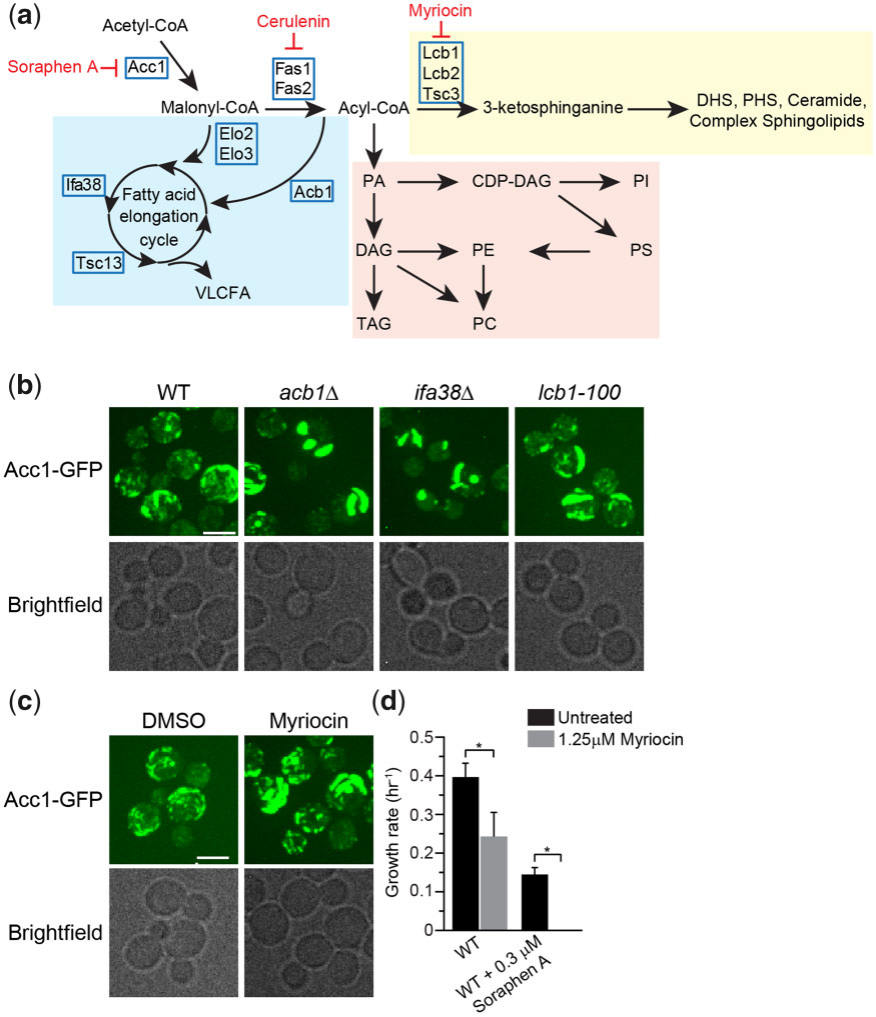
Inhibition of the FA elongation cycle and sphingolipid metabolism induces the formation of thick rod-like structures of Acc1-GFP. a) Schematic of the FA elongation and sphingolipid metabolism pathway that includes genes that encode the enzymes within pathway and pharmacological inhibitors Myriocin and Cerulenin. b) *acb1Δ*, *ifa38Δ*, and *lcb1-100* mutants impact the localization of Acc1-GFP. Wild-type (YKB 3954), *acb1Δ* (YKB 4601), *ifa38Δ* (YKB 4629), and *lcb1-100* (YKB 4609) cells expressing endogenously tagged Acc1-GFP were grown to early-log phase at 30°C in YPD and immediately assessed for Acc1-GFP localization within the cells. Representative brightfield and fluorescent images are shown. Scale bar: 4 µm. c) Myriocin, a SPT inhibitor, induces the formation of thick rod-like structures of Acc1-GFP. Wild-type (YKB 3954) cells expressing endogenously tagged Acc1-GFP were grown to early-log phase at 30°C in YPD. Cells were then again diluted to an OD_600_ of 0.1 in YPD with or without 1.25 µM Myriocin, and then grown for 3 h at 30°C before imaging Acc1-GFP localization within the cells. Representative brightfield and fluorescent images are shown. Scale bar: 4 µm. d) Myriocin significantly increases the toxicity of Sor A. Wild-type (YKB 1079) cells were grown to early-log phase diluted to an OD_600_ of 0.1 in YPD with or without 0.3 µM Sor A and/or 1.25 µM Myriocin, automated growth curve analysis was performed at 30°C for 48 h and growth rate calculated from 3 biological replicates. Error bars denote the standard error of the mean (SEM). *n* = 3, *P < 0.05 determined using a 2-way ANOVA test with a Sidak’s multiple comparisons test.

Given the remarkable impact of NuA4 on Acc1 localization, to begin to discern the biological pathways regulating Acc1-GFP localization that NuA4 may be involved in, we performed a high content microscopy screen. We employed SGA technology (Tong *et al.* 2001) to introduce Acc1-GFP into 84 deletion mutants from the BY4741 deletion mutant collection (Giaever *et al.* 2002). The deletion mutants selected encoded proteins previously implicated in Acc1 regulation including AMPK signaling, citrate metabolism, FA oxidation, sphingolipid metabolism, glucose signaling, and metabolism (Supplementary Table 1). Once constructed the Acc1-GFP mutant array strains were imaged and 5 deletion mutants impacted the localization of Acc1-GFP: *SNF1*, *SNF4*, *ELO2*, *ELO3*, and *TSC3* (Supplementary Fig. 3). While no mutants displayed the diffuse phenotype similar to *eaf1*Δ cells, as expected *snf1*Δ Acc1-GFP cells in the screen displayed large foci similar to what we found in our directed studies with *snf1Δ* Acc1-GFP and Acc1^S1157A^-GFP cells (Fig. 2a). Large foci were also detected in *snf4Δ* cells which was expected as Snf4 is the regulatory (gamma) subunit of AMPK1/Snf1 and Snf1 activity is reduced in the absence of Snf4 (Celenza *et al.* 1989). In contrast, the 3 other mutants identified in the screen displayed Acc1-GFP localized into thick curved rod-like structures that formed around the periphery of the cell. These Acc1 structures are different in shape and localization from the Acc1-GFP punctate dots seen in *snf1Δ* (Fig. 3, a and b; Supplementary Fig. 3). Intriguingly, all 3 mutants identified are involved in FA elongation and sphingolipid biosynthesis [reviewed in Erdbrügger and Fröhlich (2020) and Fig. 4a]. Elo2 and Elo3 are FA elongases in the VLCFA, biosynthesis pathway while Tsc3 is an activator of serine palmitoyltransferase (SPT), which is composed of Lcb1 and Lcb2 (Gable *et al.* 2000). We next assessed the sensitivity of these strains to Sor A to determine if these 3 mutants also potentially had changes in Acc1 activity. Even at 0.1 µM, a concentration that does not impact the growth of wt cells, Sor A was extremely toxic to *elo2Δ*, *elo3*Δ, and *tsc3*Δ cells suggesting they have low levels of Acc1 activity (Fig. 3c). Our results confirm high-throughput Sor A chemogenomic screens which identified these mutants as well as work identifying impaired FA elongation in myriocin tolerance adaptation (Hoepfner *et al.* 2014; Bozaquel-Morais *et al.* 2017; Randez‐Gil *et al.* 2020). A reduction in Acc1 activity upon deletion of *ELO3* was further confirmed using an *in vitro* assay to measure the activity of Acc1 purified from *elo3Δ* cells (Fig. 3d).

As 2 of these mutants were implicated in the VLCFA cycle, we next directly assessed Acc1-GFP localization in nonessential VLCFA cycle genes that were not included in our screen (Fig. 4a). Acb1 transports newly synthesized acyl-CoA esters from FA synthesis into the VLCFA biosynthesis pathway (Schjerling *et al.* 1996), while Ifa38 is a reductase that reduces 3-ketoacyl-CoA to 3-hydroxyacyl-CoA (Han *et al.* 2002). Like *elo3Δ* and *elo2Δ*, deletion of *ACB1* and *IFA38* caused a thick Acc1-GFP rod formation to occur (Fig. 4b). Similarly, as the third mutant, *tsc3Δ*, has reduced SPT activity, we extended our study to assess Acc1-GFP localization in a temperature-sensitive point mutant of *LCB1*, *lcb1-100*, 1 of 2 essential catalytic subunits of SPT (Sütterlin *et al.* 1997). In parallel, we also assessed the effect of myriocin, a SPT inhibitor (Miyake *et al.* 1995), on Acc1-GFP localization and Sor A sensitivity. Both conditions resulted in the formation of thick rod structures (Fig. 4, b and c) and myriocin treatment significantly increases the toxicity of Sor A, suggesting reduction in SPT activity decreases Acc1 activity in cells (Fig. 4d). Taken together, we have determined that inhibition of VLCFA elongation cycle and SPT results in the concentration of Acc1-GFP into thick rod-like structures which is associated with impaired Acc1 activity.

### Palmitic acid induces Acc1-GFP rod formation and inhibits Acc1 activity

**Fig. 5.**
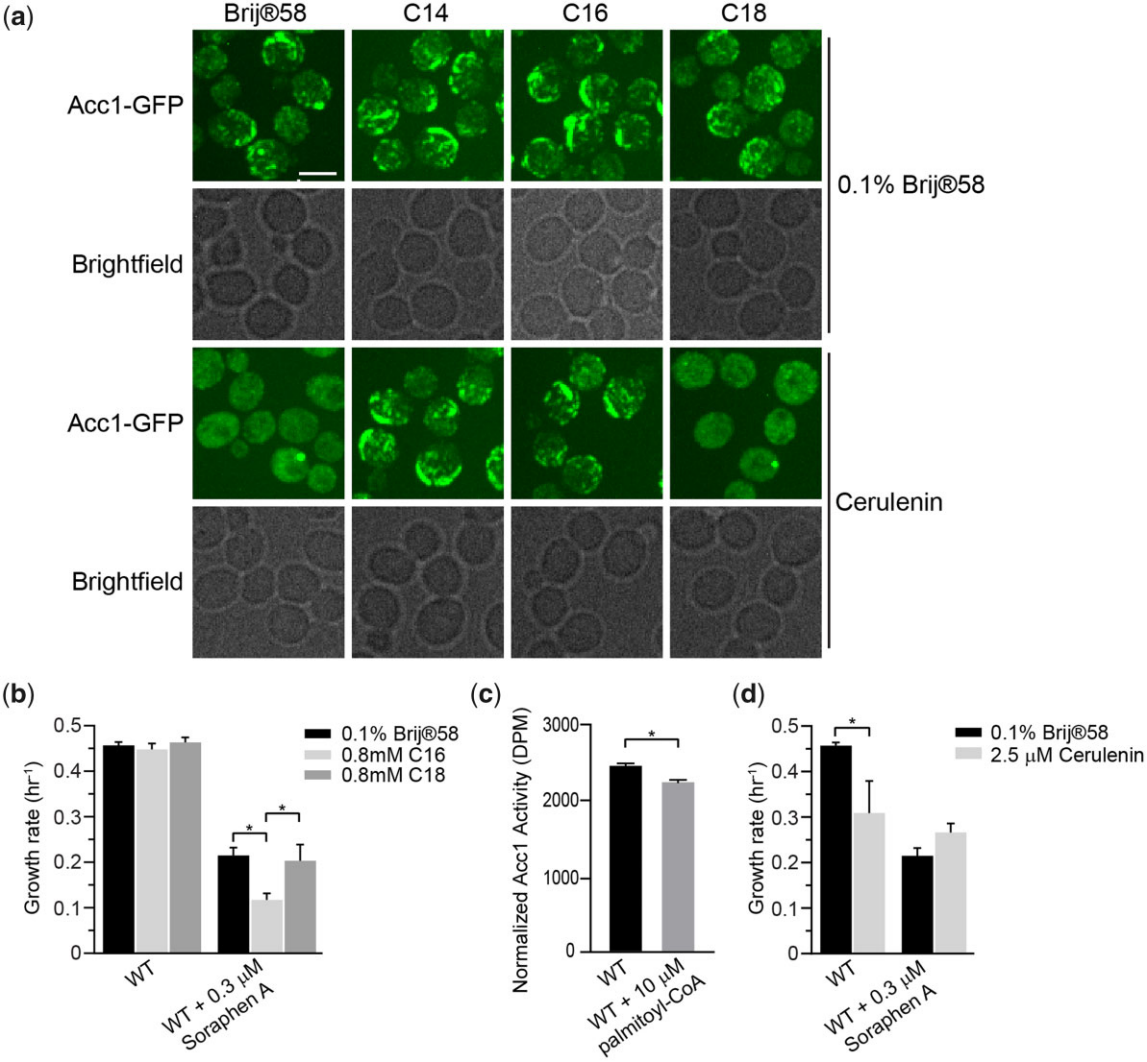
Palmitic acid induces the formation of thick rod-like structures of Acc1-GFP and inhibits Acc1 activity. a) Wild-type (YKB 3954) cells expressing endogenously tagged Acc1-GFP were grown to early-log phase before being diluted to an OD_600_ of 0.1 in YPD containing 0.1% Brij 58 with or without 0.8 mM myristic acid (C14), 0.8 mM palmitic acid (C16), or 0.8 mM stearic acid (C18) and/or 40 µM Cerulenin, grown for 1 h at 30°C and immediately assessed for Acc1-GFP localization. Representative brightfield and fluorescent images are shown. Scale bar: 4 µm. b) Palmitic acid (C16) increases sensitivity of wt cells to Sor A. Wild-type (YKB 1079) cells were grown to early-log phase at 30°C in YPD. Cells were diluted to an OD_600_ of 0.1 in YPD containing 0.1% Brij 58 with or without 0.3 µM Sor A and/or 0.8 mM palmitic acid (C16) or 0.8 mM stearic acid (C18), automated growth curve analysis at 30°C for 48 h and growth rate was then calculated from 3 biological replicates. Error bars denote the standard error of the mean (SEM). *n* = 3, *P < 0.05 determined using a 2-way ANOVA test with a Tukey’s multiple comparisons test. c) Wild-type (YKB 3954) cells expressing endogenously tagged Acc1-GFP were grown to early-log phase at 30°C in YPD. Acc1-GFP was then immunoprecipitated and incubated with or without 10 µM Palmitoyl-CoA prior to measure it’s specific activity as described in the *Materials and Methods*. Acc1-GFP activity was normalized to the relative protein abundance in the sample. Three biological replicates were performed. Error bar indicates the standard error of the mean (SEM). * Denotes statistical significance at a *P*-value < 0.05 determined using a *t*-test. d) Cerulenin does not increase sensitivity of wt cells to Sor A. Wild-type (YKB 1079) cells were grown to early-log phase at 30°C in YPD. Cells were diluted to an OD_600_ of 0.1 in YPD containing DMSO with or without 0.3 µM Sor A and/or 2.5 µM Cerulenin, and an automated growth curve analysis was performed at 30°C for 48 h and the growth rate was then calculated from 3 biological replicates. Error bars denote the standard error of the mean (SEM). *n* = 3, *P < 0.05 determined using a 2-way ANOVA’s test with a Sidak’s multiple comparisons test.

Remarkably, the mutants that displayed rod-like localization of hypoactive Acc1-GFP, along with myriocin treatment (Fig. 4), share the common feature that their disruption leads to increased cellular acyl-CoAs such as palmitoyl-CoA. Furthermore, exogenous long-chain FAs have been shown to inhibit Acc1 activity in yeast extracts (Kamiryo *et al.* 1976; Shirra *et al.* 2001) and 16–20 carbon long-chain length acyl-CoAs directly inhibit rat liver Acc1 (Ogiwara *et al.* 1978; Nikawa *et al.* 1979). Therefore, we next sought to determine if the formation of Acc1-GFP rod-like structures could be driven by an increase in acyl-CoA or FAs levels. We first asked if exogenous treatment of cells with myristic acid (C14), palmitic acid (C16), or stearic acid (C18), which are converted to their respective CoA derivatives, impacts Acc1-GFP localization. Brij 58 was used as a vehicle control and had no impact on Acc1-GFP localization, but treatment with myristic acid (C14) and palmitic acid (C16) induced Acc1-GFP rods (Fig. 5a). In contrast stearic acid (C18) treatment did not induce Acc1-GFP rod formation. In agreement with the correlation that Acc1-GFP rod formation is indicative of decreased Acc1 activity, cells treated with palmitic acid (C16) had an increased sensitivity to Sor A compared to untreated or stearic acid (C18) treated cells (Fig. 5b). A small but significant decrease in the *in vitro* Acc1 activity in the presence of palmitoyl-CoA was also identified (Fig. 5c). This suggests that chain length of acyl-CoA and downstream FAs and not just concentration impacts Acc1-GFP localization and activity. As increased short (C14–C16) acyl-CoAs induced Acc1-GFP rods and inhibit Acc1 activity, we next asked whether a decrease in these acyl-CoAs impacts Acc1-GFP localization and activity by treating the cells with cerulenin, an inhibitor of FA synthesis (Vance *et al.* 1972). Surprisingly, we detected diffuse localization of Acc1-GFP upon cerulenin treatment which could be suppressed by exogenous myristic acid (C14) and palmitic acid (C16), but not stearic acid (C18) (Fig. 5a). Though the diffuse localization of Acc1-GFP upon cerulenin treatment is reminiscent of *eaf1*Δ cells (Fig. 2), cerulenin treatment decreased the sensitivity of wt cells to Sor A treatment (Fig. 5d). This suggests that inhibiting FAS activity, thereby reducing acyl-CoA synthesis, impacts the localization, and enhances the activity of Acc1, which can be reversed by exogenous supply of C14–C16 FA. Together our work suggests that not only is Acc1 activity negatively regulated by short acyl-CoAs, but that short acyl-CoA chains (C14–C16) can influence the localization of Acc1-GFP.

### Cerulenin treatment partially suppresses Sor A sensitivity of *eaf1Δ* cells which have elevated FAS activity

**Fig. 6.**
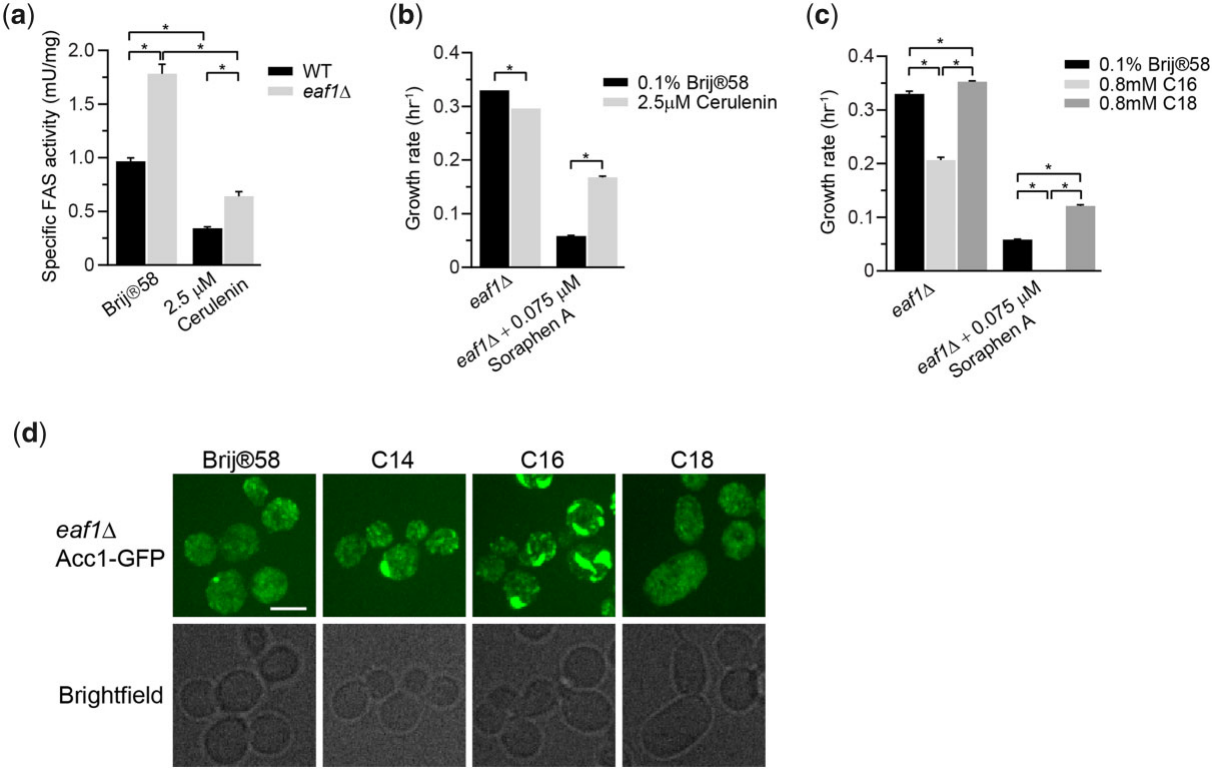
Cellular acyl-CoA chain length and FAS activity partially modulate *eaf1Δ* cell sensitivity to Sor A. a) FAS activity is increased in *eaf1Δ* cells compared to wt cells, but Cerulenin inhibits FAS activity in both cells. Wild-type (YKB 1079) and *eaf1Δ* (YKB 3333) were grown to early-log phase at 30°C in YPD. Cells were then again diluted to an OD_600_ of 0.1 in YPD with or without 2.5 µM Cerulenin, and then grown for 2 h at 30°C. FAS activity assay was performed on the whole-cell extracts and was normalized to the relative protein abundance in the sample. Error bars denote the standard error of the mean (SEM). *n* = 3, *P < 0.05 determined using a 2-way ANOVA test with a Tukey’s multiple comparisons test. b) Cerulenin partially suppresses sensitivity of *eaf1Δ* cells to Sor A. *eaf1Δ* (YKB 4448) cells were grown to early-log phase at 30°C in YPD. Cells were then diluted to an OD_600_ of 0.1 in YPD with or without 0.075 µM Sor A and/or 2.5 µM Cerulenin, and an automated growth curve analysis was performed and used to calculate growth rate. Error bars denote the standard error of the mean (SEM). *n* = 3, *P < 0.05 determined using a 2-way ANOVA test with a Sidak’s multiple comparisons test. c) Palmitic acid (C16) increases sensitivity of *eaf1Δ* cells to Sor A. *eaf1Δ* (YKB 4448) cells were grown to early-log phase before being diluted to an OD_600_ of 0.1 in YPD containing 0.1% Brij 58 with or without 0.075 µM Sor A and/or 0.8 mM palmitic acid (C16) or 0.8 mM stearic acid (C18), automated growth curve analysis was performed at 30°C for 48 h and growth rate was then calculated from 3 biological replicates. Error bars denote the standard error of the mean (SEM). *n* = 3, *P < 0.05 determined using a 2-way ANOVA test with a Sidak’s multiple comparisons test. d) Palmitic acid induces the formation of thick rod-like structures of Acc1-GFP in *eaf1Δ* cells. *eaf1Δ* (YKB 4448) cells expressing endogenously tagged Acc1-GFP were grown to early-log phase before being diluted to an OD_600_ of 0.1 in YPD containing 0.1% Brij 58 with or without 0.8 mM myristic acid (C14), 0.8 mM palmitic acid (C16), or 0.8 mM stearic acid (C18) and grown for 2 h at 30°C prior to imaging. Representative brightfield and fluorescent images are shown. Scale bar: 4 µm.

Given the profound impacts of C14–C16 acyl-CoA and FAS activity on Acc1 localization and activity, we next tested if NuA4 regulates Acc1 via this pathway. First, we assessed FAS activity in *eaf1Δ* cells. In agreement with transcriptome studies which have identified an induction of *FAS1* and *FAS2* mRNA in *eaf1Δ* background (Dacquay *et al.* 2017), Fas1 and Fas2 protein levels are increased in *eaf1Δ* cells (Supplementary Fig. 4). FAS activity is also significantly increased nearly doubling in *eaf1Δ* cells compared to wt cells, which is decreased by Cerulenin treatment to inhibit FAS activity (Fig. 6a). Furthermore, we found that cerulenin treatment to inhibit FAS partially suppressed Sor A sensitivity of *eaf1Δ* cells (Fig. 6b) while exogenous palmitic acid (C16) treatment reduced the growth rate of *eaf1Δ* cells and increased the sensitivity of *eaf1Δ* cells to Sor A (Fig. 6c). The hypersensitivity of palmitic acid (C16) treated *eaf1Δ* to Sor A is consistent with the formation of large rod structures (Fig. 6d) previously shown to correlate with reduced Acc1 activity. Interestingly, stearic acid (C18) partially suppressed Sor A sensitivity. Together this suggests that despite puzzlingly similar diffuse Acc1-GFP localization in *eaf1Δ* and cerulenin treated cells, elevated FAS activity and its products C14–C16 acyl-CoAs are indeed contributing to a reduction of Acc1 activity and increased Sor A sensitivity in *eaf1Δ* cells in a negative feedback loop.

### Lipids are overall deregulated in NuA4 mutants

**Fig. 7.**
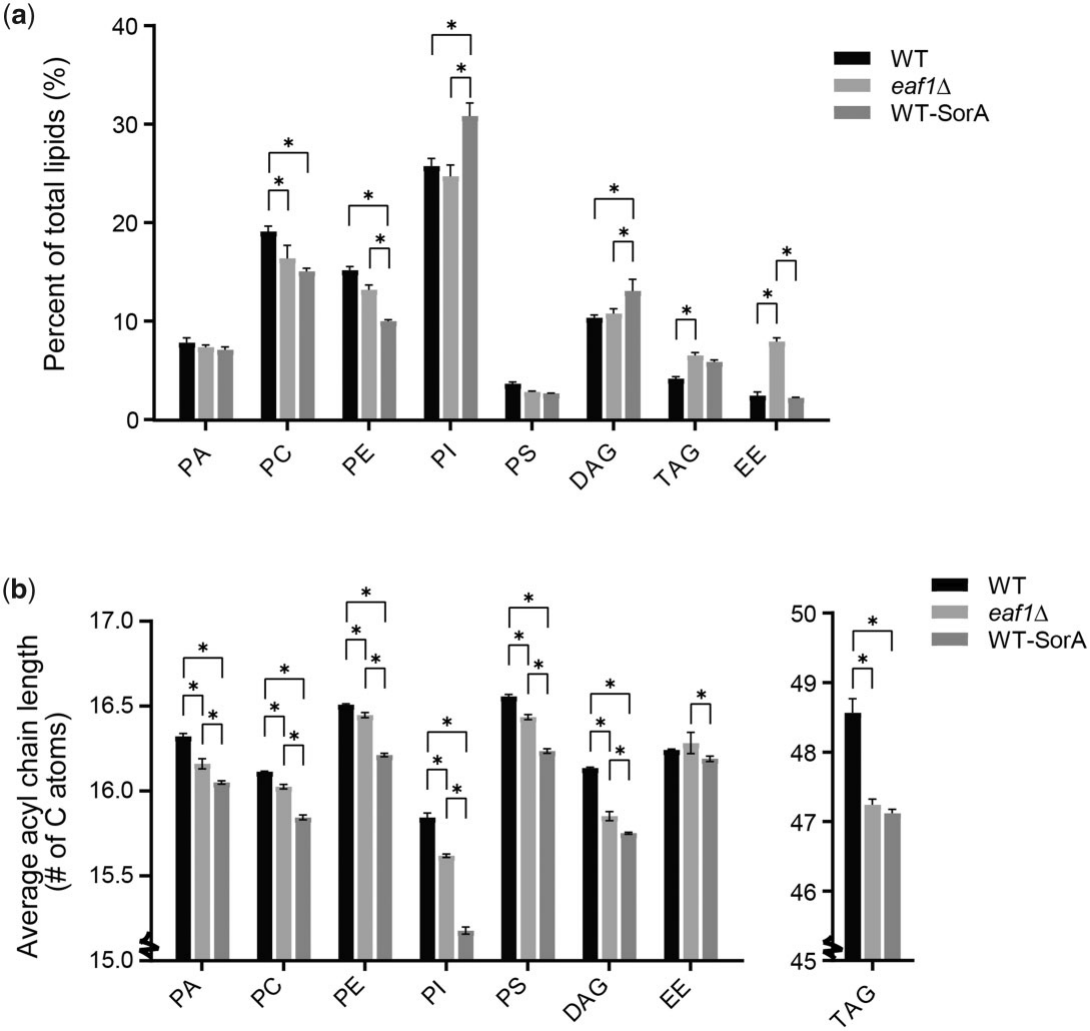
Lipidomic analysis of WT and NuA4 mutants. The lipid composition of WT, *eaf1Δ*, and Sor A treated WT yeast was assessed through lipidomics. WT, *eaf1Δ*, and Sor A treated cells were harvested in mid-log and a crude lysate was created through bead beating with water, lipids were then extracted using a chloroform/methanol procedure and Mass spec analysis was performed by Lipotype GmbH. In this figure are the most prevalent lipids identified: PA, Phosphatidylcholine (PC), Phosphatidylethanolamine (PE), Phosphatidylinositol (PI), Phosphatidylserine (PS), Diacylglycerol (DAG), Ergosteryl ester (EE), and Triacylglycerol (TAG). a) The composition of lipid classes broken down as a percent of the total lipids. A 2-way ANOVA analysis with a Tukey’s multiple comparisons test was performed and a * represents an adjusted *P* < 0.05. b) The average acyl chain length for each class of lipid was calculated from the lipidomic data. The *Y*-axis is broken to zoom in on the data and show the small but significant changes in chain length. A 2-way ANOVA analysis with a Tukey’s multiple comparisons test was performed and a * represents an adjusted *P* < 0.05.

Our data are consistent with the findings of multiple studies that not only is Acc1 regulating acyl-chain length, but that acyl-chain length is directly impacting Acc1 activity through a feedback loop. Hyperactive Acc1 does not significantly change the composition of membrane phospholipids, but leads to an increase in total TAG and a shift toward longer acyl-chain lengths across the glycerolipid classes (Hofbauer *et al.* 2014). Conversely, Acc1 hypomorphs and Sor A treated cells also do not dramatically alter most phospholipid composition, but display a shift to shorter average acyl chain length (Schneiter *et al.* 2000; Bao *et al.* 2021). Furthermore, increased FAS activity, which increases cellular concentration of acyl-CoA also directly inhibits Acc1 activity (Kamiryo *et al.* 1976; Ogiwara *et al.* 1978). In addition, our work suggests that acyl chain length is also influencing the subcellular localization and activity of Acc1. This negative feedback seems to partially account for the reduction of Acc1 in the NuA4 mutant *eaf1*Δ. To further characterize the impacts of NuA4 on Acc1 via lipid metabolism in general and through acyl chain length in particular, we performed a shotgun lipidomic analysis to look for overall cellular lipid profile changes in WT, *eaf1Δ*, and Sor A treated cells (Fig. 7 and Supplementary Table 3). Similar to previous studies, decreasing Acc1 activity through Sor A treatment resulted in minor changes in neutral lipid composition of cells, including increased PI and decreases in PC (Bao *et al.* 2021). The limited impact of Sor A on PA and even TAG levels indicates that the cell is able to compensate for overall changes in neutral lipids to maintain homeostasis as previously shown for hyperactive Acc1 mutants (Hofbauer *et al.* 2014). More striking was the common characteristic of a reduction in average number of carbons in the tail chain length for all neutral lipid species in cells treated with Sor A (Fig. 7). Interestingly, while deletion of *EAF1* also did not show broad changes in the composition of the lipotype by lipid class, it also had the trend of decreased average acyl chain length although to a slightly less degree than the Sor A treated condition (Fig. 7). The one significant difference between *eaf1Δ* and Sor A treated cells is that the percentage of ergosterol esters was increased in *eaf1Δ* cells, which suggests NuA4 may be playing a separate role in ergosterol metabolism. Together this work shows that similar to *acc1* hypomorphs, *eaf1Δ* cells display a reduction in acyl chain length.

## Discussion

**Fig. 8.**
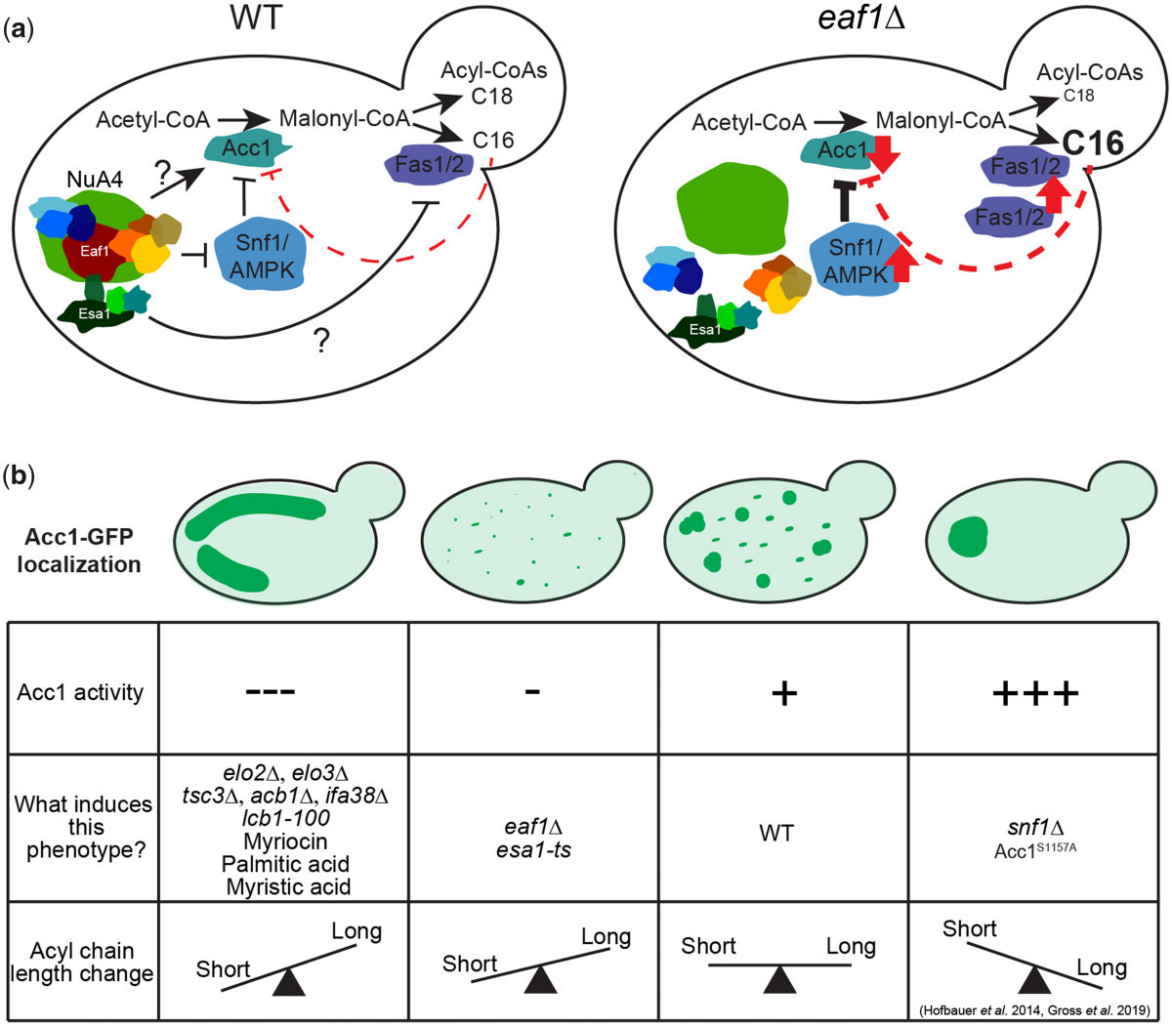
Model for NuA4-dependent regulation of Acc1. a) Schematic representing the regulation of Acc1 in WT and *eaf1Δ* yeast by NuA4. b) Four different phenotypes of Acc1-GFP localization were identified in our study. These phenotypes are demonstrated along with a table summarizing the activity of Acc1, causes of the phenotype, and the pattern of acyl-CoA chain length associated with the each of the phenotypes.

Through multiple biochemical, chemical, and genetic approaches, we confirm that Acc1 activity is reduced upon deletion of *EAF1* and disruption of the NuA4 complex (Rollins *et al.* 2017) is associated with more diffuse subcellular localization of Acc1-GFP. We determined that in addition to Eaf1, multiple pathways impact Acc1 subcellular localization and activity, including increased palmitoyl-CoA (C16) levels which inhibits Acc1 activity and remodels Acc1 localization. Finally, we found that *eaf1Δ* cells do not have drastic changes in the composition of cellular lipids by class but that they did appear to have reduced overall chain length across FAs, which phenocopies Acc1 inhibition through Sor A treatment. Therefore, in addition to the well-known Snf1/AMPK signaling pathway, NuA4 regulation of Acc1 may be through a dynamic feedback mechanism in which altered lipid length can impact Acc1 localization and activity (Fig. 8a).

### Multifaceted regulation of Acc1 by NuA4

Given the established role of NuA4 in negatively regulating Snf1/AMPK (Lu *et al.* 2011), we anticipated that the decrease in Acc1 activity in *eaf1Δ* cells would be solely due to increased inhibition by Snf1/AMPK phosphorylation. If NuA4 was only inhibiting Acc1 due to hyperactive Snf1, we would anticipate that deletion of *SNF1* in an *eaf1Δ* background would result in hyperactive Acc1 and that *snf1Δ* and *snf1Δeaf1Δ* cells would have similar resistance to Sor A treatment. Surprisingly, we found this was not the case and our work suggests that NuA4 is regulating Acc1 through both Snf1-dependent and independent pathways (Fig. 1). As NuA4 directly regulates other metabolic enzymes (Lin *et al.* 2009; Lu *et al.* 2011), a likely mechanism for the secondary pathway could be direct acetylation of Acc1. However, though global acetylome studies have identified multiple acetylation sites on Acc1, to date none are attributed to NuA4 (Henriksen *et al.* 2012; Weinert *et al.* 2014; Downey *et al.* 2015; Madsen *et al.* 2015). Nor did our SILAC-based proteomic study identify any acetylation sties on Acc1-TAP that are dependent on the catalytic subunit of NuA4, *esa1* (Supplementary Fig. 1). Though we cannot definitely rule out that NuA4 is regulating the activity of Acc1 through direct acetylation, which would require a systematic mutational analysis of all potential lysine acetylation sites on Acc1 and assessment of activity* in*v*ivo*, our work suggests that NuA4 is likely regulating Acc1 through a more complex mechanism that includes regulation of subcellular localization of Acc1.

### Correlation between Acc1 localization and activity

Even as early as the 1970s, it was reported that ACCs from various species form filaments and this polymerization facilitates activation of the enzyme (Mackall *et al.* 1978; Meredith and Lane 1978; Beaty and Lane 1983). Localization studies of Acc1 in yeast have also indicated that Acc1 can form aggregates. Under logarithmic growth conditions, both indirect immunofluorescence and live GFP imaging have shown that Acc1 is largely diffuse with small punctate structures across the cytoplasm (Schneiter *et al.* 1996; Ivessa *et al.* 1997; Wolinski *et al.* 2009). More recent studies have shown that Acc1 can form rod-like structures in the center of the cell in 19% of cells in log phase and these central rod-like filaments increase to 57% cells after 5 days of starvation (Noree *et al.* 2019). Other directed and high-content screens have also detected dramatic changes in Acc1 subcellular location upon stress including oxygen deficiency, rapamycin treatment, and DTT treatment (Wolinski *et al.* 2009; Breker *et al.* 2013; Chong *et al.* 2015). One screen showed a change from a cytosolic punctate localization to aggregate filament structures similar to those seen in our work upon treatment of Acc1-GFP cells with DTT (Breker *et al.* 2013). However, to our knowledge, the diffuse localization of Acc1-GFP that we found in the *eaf1Δ* cells and the Acc1 localization of rod-like structures around the periphery of the cell have not yet been documented in yeast.

The association between the changes in localization and activity of Acc1-GFP in each of the mutants was a key finding in our work. We found that in mutants with high Acc1 activity, such as *snf1Δ* and *acc1^S1157A^* mutants, Acc1-GFP was highly aggregated into a small number of large rod or punctate structures within the center of the cell (Fig. 8b). Wild-type levels of Acc1 activity were associated with many punctate structures throughout the cell. Mutants with slightly lowered Acc1 activity, such as our NuA4 mutants displayed decreased aggregation of Acc1 resulting in a more diffuse distribution. Interestingly, our understanding that NuA4 regulation of Acc1 may be independent of Snf1 activity was additionally supported by the fact that the Acc1-GFP aggregates were smaller in the *eaf1Δ snf1Δ* and *eaf1Δ acc1^S1157A^* mutants than in the *snf1Δ* and acc1^*S1157A*^ mutants. However, unlike NuA4 mutants, mutants with very low Acc1 activity, such as those involved with sphingolipid metabolism or VLCFA elongation, demonstrated Acc1 aggregation into large rod-like structures, but these were predominantly visible toward the periphery of the cell (Fig. 8b). The shape and localization of these inactive Acc1 rods are different from the ones formed by hyperactive Acc1 in *snf1Δ* and *acc1^S1157A^* mutants.

While in yeast, the correlation of Acc1 activity and localization/aggregation has not previously been explored, recent work by Hunkeler and colleagues obtained cryo electron microscopy structures of human Acc1 filaments which were associated with Acc1 activity. These filaments formed in the presence of citrate (with and without palmitoyl CoA) and the BRCT domains of BRCA1 (Hunkeler *et al.* 2018). Interestingly, these filaments were associated with high and low levels of Acc1 activity, respectively, and the high activity of the citrate-associated filament was inhibited by the addition of excess palmitoyl-CoA, somewhat mirroring the effects of palmitic acid in our work (Hunkeler *et al.* 2018). However, while we have previously shown that citrate can impact Acc1 activity *in vitro*, an overlay of the yeast Acc1 protein onto the determined human ACC1 filament suggests that the yeast Acc1 is incompatible with the aggregation structures identified by Hukeler *et al.* (Kim *et al.* 2016; Rollins *et al.* 2017; Hunkeler *et al.* 2018). Furthermore, while the addition of citrate to Acc1 extracted from *eaf1Δ* yeast did slightly improve activity, it was well below that of WT, suggesting an alternate mechanism of Acc1 regulation by NuA4 (Rollins *et al.* 2017). While our yeast aggregates may not form the filaments documented in the mammalian system, we show the formation of Acc1 aggregates is associated with both low (periphery rods) and high levels (internal large foci) of Acc1 activity and that these structures do appear as separate aggregate phenotypes.

To complicate things, we found that an inhibition of FAS led to a diffuse Acc1 similar to that of the *eaf1Δ* localization, but this treatment seemed to make the cells less sensitive to Sor A. This suggests that in contrast to *eaf1Δ*, under FAS inhibition Acc1 activity is increased despite the largely diffuse localization. One possible explanation for this inconsistency is that even though seemingly similar, the diffuse Acc1 can exist in 2 different states or microstructures that associate with high or low activity, a phenomenon observed with the Acc1 rod-like structures and filaments as discussed above. In fact, we did observe the Acc1 structures similar to those in *snf1Δ* and *acc1^S1157A^* mutants in a few cells under FAS treatment. Our observation raises the possibility that Acc1 localization is highly dynamic and the diffusion of Acc1 structures may represent intermediate states where Acc1 transitions from one type of aggregate to another. Further time-lapse studies using higher imaging resolution may be needed to prove this hypothesis.

The change in localization associated with changes in Acc1 activity in our yeast could have many impacts including impacting protein interactions of Acc1. In fact, a screen that previously identified Acc1 aggregation into rods upon DTT treatment compared the protein interactors of untreated and treated cells and identified 9 new physical interactors proteins of Acc1 upon DTT treatment (Breker *et al.* 2013). However, Noree *et al.*, the authors who identified Acc1 rods under glucose limitation did not identify any aggregating proteins that colocalized with Acc1 structures. While this does not rule out that there are other lower abundance proteins, lipids, or other compounds present in the Acc1 aggregates, it suggests that the primary protein component of these structures under these conditions is Acc1 (Noree *et al.* 2019). In the future, it will be interesting to determine if the different types of aggregates demonstrated in our work, with high and low Acc1 activity and central vs peripheral localization, respectively, are associated with different compositions.

### Feedback of FAS activity and inhibition of Acc1 through shorter chain acyl-CoAs

After our screen for mutants that affect Acc1-GFP localization highlighted the importance of VLCFA and sphingolipids in maintaining Acc1 localization and activity (Figs. 3 and 4), we asked if acyl-CoA feedback inhibition on Acc1 (Kamiryo *et al.* 1976; Ogiwara *et al.* 1978; Nikawa *et al.* 1979; Shirra *et al.* 2001), could be mediated by regulation of Acc 1 subcellular localization.

We found that the effect of the acyl-CoA treatment on Acc1-GFP localization was dependent on the chain length, with the shorter myristic acid (C14) and palmitic acid (C16) inducing rods but not the longer stearic acid (C18) (Figs. 5 and 6). Treatment of cells with C16 also increased their sensitivity to Sor A, indicating a decrease in Acc1 activity upon this treatment. The addition of shorter chain length FAs having a phenotypic effect aligns well with previous research linking Acc1 activity, FAS activity, and acyl-CoA chain lengths but here we identify an additional change in Acc1 localization (Hofbauer *et al.* 2014; Gross *et al.* 2019; Randez‐Gil *et al.* 2020; Bao *et al.* 2021).

Independent studies by Gross and Hofbauer found that inositol auxotrophy and autophagy defects of the hyperactive mutant Acc1^S1157A^ are phenocopied by adding a longer chain FA, Oleic acid (C18) (Hofbauer *et al.* 2014; Gross *et al.* 2019). In fact, exogenous treatment with Oleate was able to partially reverse an autophagy defect found in Sor A treated cells, suggesting that longer chain acyl-CoAs could feed back onto regulation of Acc1 activity (Hofbauer *et al.* 2014; Gross *et al.* 2019). Furthermore, the hyperactive Acc1^S1157A^ mutant has an overall shift in lipid content toward longer chain FAs with an increase in C18/C16 ratio and an increase in average chain length of FAs (Hofbauer *et al.* 2014; Gross *et al.* 2019). Similarly, recent work by Bao *et al.* (2021) identified a Acc1 hypomorph that allowed growth in the absence of PC. This evolution was associated with a decrease in Acc1 activity and acyl-CoA chain length. Intriguingly, this study also found that a chromosome XV monosomy allowed yeast to grow in the absence of PC (Bao *et al.* 2021). This mutant had apparent increase in FAS activity that was associated with a decrease in acyl-CoA chain length and an inhibition of Acc1 (Bao *et al.* 2021). In agreement with this finding, our work shows a small but significant decrease in lipid chain length in our *eaf1Δ* cells and in Sor A treated cells which coincided with an increase in FAS activity and decrease in Acc1 activity. Additionally, an inhibition of FAS alleviated sensitivity to Sor A in both WT and *eaf1Δ* mutant, suggesting that Acc1 activity is increased under FAS inhibition. Intriguingly, the catalytic subunit of NuA4, *ESA1*, is found on chromosome XV, which raises the possibility that the chromosome XV monosomy that suppresses PC-deficiency (Bao *et al.* 2021) could partially be through an *ESA1* haploinsufficiency. The decreased Acc1 activity and the reduction in lipid chain length upon deletion of *EAF1* that we report here parallels the increase in chain length in the hyperactive Acc1^S1157A^ by Hofbauer and colleagues as well as the decrease in the chain length in hypoactive Acc1 conditions by Bao and colleagues (Gross *et al.* 2019). Finally, we were also able to demonstrate that the addition of palmitoyl-CoA decreases Acc1 activity in an *in vitro* assay (Fig. 5c), thereby suggesting that the decrease in average chain length identified in the *eaf1Δ* may play a role in the indirect regulation of Acc1 activity. The combination of our work with previously published studies suggests that not only does the activity of ACC and FAS impact the production and length of FAs but conversely the FA composition, specifically in terms of length, can affect the activity of Acc1 and that this regulation is additionally entangled with the changes in Acc1 localization reported in our study.

### Conclusions

Our work demonstrates that NuA4 is required for Acc1 activity and localization, as NuA4 mutants have a decrease in Acc1 activity which is correlated with a diffuse localization of Acc1. We also identify other discrete localizations of Acc1 which are associated with altered activity, specifically for mutants and inhibitors associated with VLCFA and sphingolipid synthesis. The identification of these mutants allowed us to implicate acyl-CoA chain length as an important factor in *EAF1* dependent regulation of Acc1. Though the exact molecular mechanism remain to be determined, our work support that deregulation of lipid length in NuA4 mutants leads to a feedback regulation on Acc1 which is correlated to changes in Acc1 localization. Our observations also highlight the complicated dynamic changes in Acc1 localization in response to cellular acyl-CoA chain length among other signals. The precise mechanism and role of yeast NuA4, and perhaps the homologous human Tip60 complex, in regulating the activity of Acc1 through lipid distributions will be an important exploration in future work.

## Data availability

All relevant data are within the manuscript and its Supporting Information files.

Supplemental material is available at *GENETICS* online.

## Supplementary Material

iyac086_Figure_S1

iyac086_Figure_S2

iyac086_Figure_S3

iyac086_Figure_S4

iyac086_Supplemental_Figure_Legends

iyac086_Table_S1

iyac086_Table_S2

iyac086_Table_S3
