# Supplementary figures and images for: Fine-tuning acetyl-CoA carboxylase 1 activity through localization: functional genomics reveals a role for the lysine acetyltransferase NuA4 and sphingolipid metabolism in regulating Acc1 activity and localization
Source: Genetics. 2022 May 24;221(4):iyac086. doi: 10.1093/genetics/iyac086 (PMC9339284; doi:10.1093/genetics/iyac086)

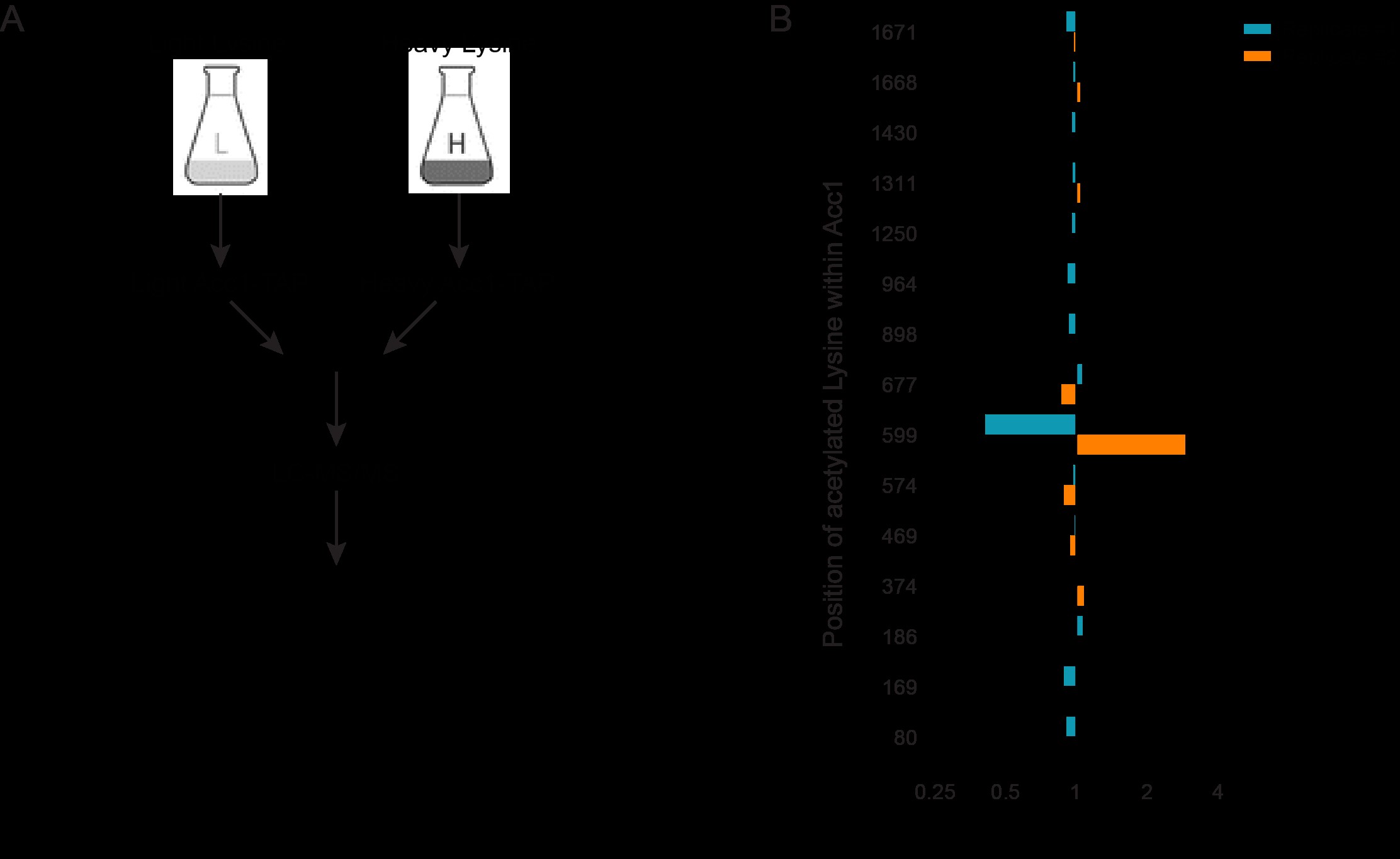

Supplement: iyac086_Figure_S1 [file iyac086_figure_s1.jpeg]

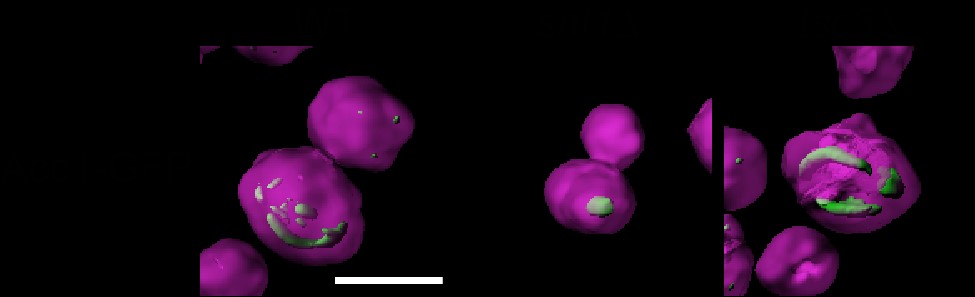

Supplement: iyac086_Figure_S2 [file iyac086_figure_s2.jpeg]

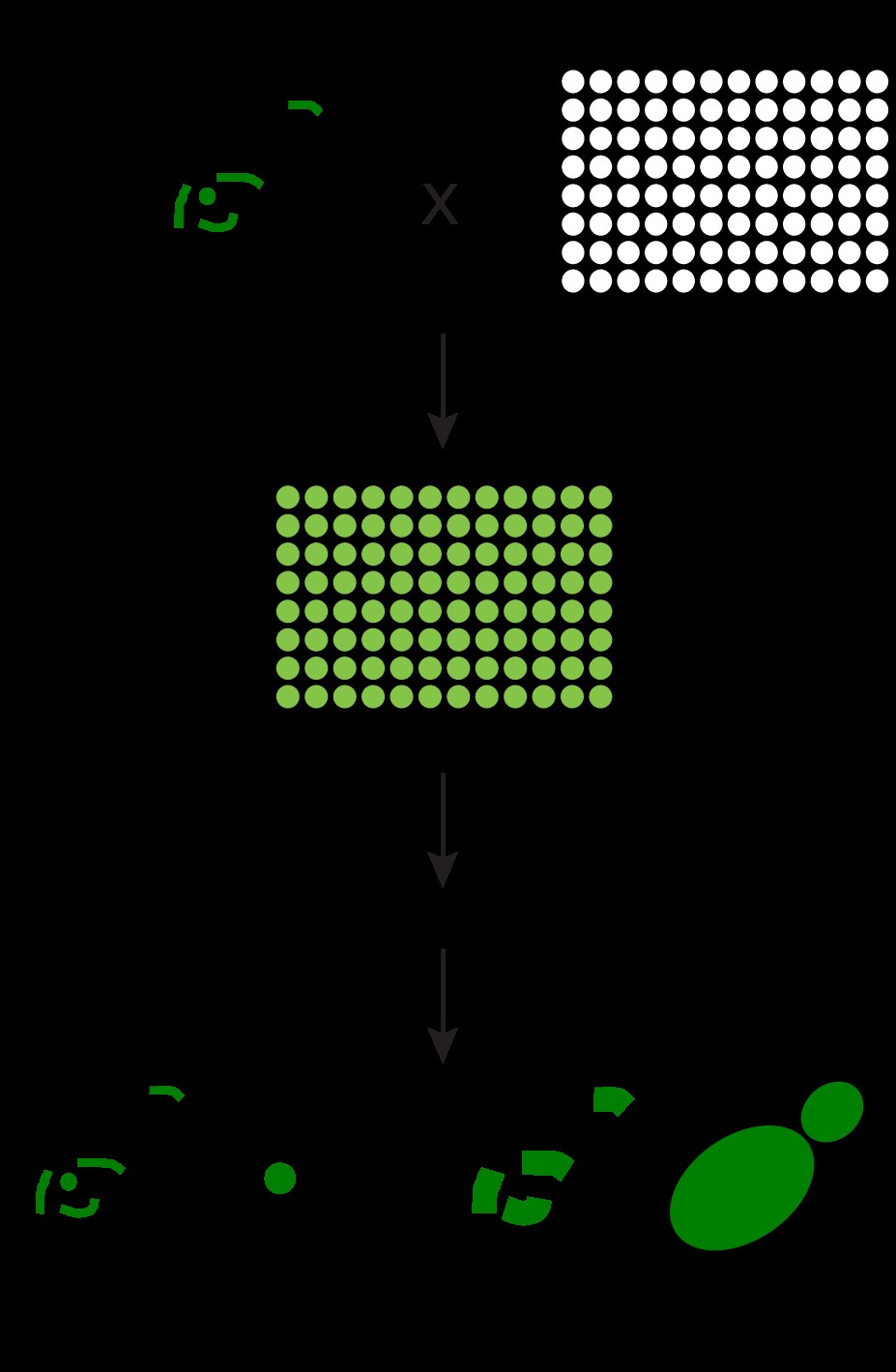

Supplement: iyac086_Figure_S3 [file iyac086_figure_s3.jpeg]

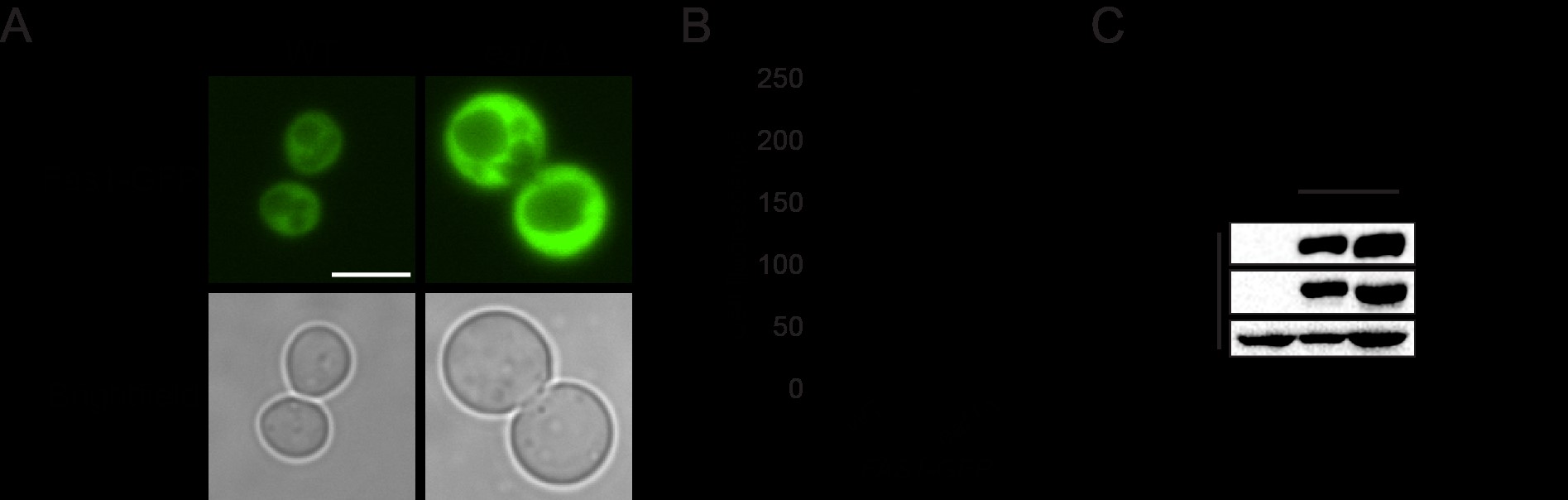

Supplement: iyac086_Figure_S4 [file iyac086_figure_s4.jpeg]
