# Supplementary material for: Fine-tuning acetyl-CoA carboxylase 1 activity through localization: functional genomics reveals a role for the lysine acetyltransferase NuA4 and sphingolipid metabolism in regulating Acc1 activity and localization
Source: Genetics. 2022 May 24;221(4):iyac086. doi: 10.1093/genetics/iyac086 (PMC9339284; doi:10.1093/genetics/iyac086)
Supplement: iyac086_Supplemental_Figure_Legends [file iyac086_supplemental_figure_legends.docx]

**Supplemental Figure Captions**

Figure S1: A) Experimental design of the SILAC, a quantitative approach to identify acetylated lysine onto immunopurified Acc1-TAP. To examine potential acetylation of lysine residues, Wild-type (YKB 4305) and *esa1-ts* (YKB 4306) expressing endogenously tagged Acc1-TAP was grown separately in heavy or light labelled lysine as described in the Materials and Methods. The heavy lysine has a mass shift of +6 Da compared to the light lysine. Acc1-TAP was immunopurified prior to digestion with trypsin. Peptides were analyzed by mass spectrometry. B) Data showing the position of the acetylated lysine within Acc1-TAP. Two biological replicates were performed.

Figure S2: A) Wild-type (YKB 3954), *eaf1Δ* (YKB 4448), *snf1 Δ* (YKB 4348), *tsc3 Δ* (YKB 4599) cells expressing endogenously tagged Acc1-GFP were grown to early-log phase at 30°C in YPD and immediately assessed for Acc1 localization within the cells. 3D reconstruction of the different Acc1-GFP structures being identified was built using Imaris image analysis software. Scale bar: 7µm.

Figure S3: The microscopic screen used to identify genetic deletions that affect Acc1 localization within the cells.

Figure S4: A) Level of Fas1-GFP is increased in *eaf1Δ.* Wild-type (YKB 3983) and *eaf1Δ* (YKB 4051) cells expressing endogenously tagged Fas1-GFP were grown to early-log phase at 30°C in YPD and immediately assessed for Fas1-GFP localization within the cells. Representative brightfield and fluorescent images. Scale bar: 5 µm. B) Mean cell GFP fluorescence was measured. Three biological replicates were performed. Error bar indicates the standard error of the mean (SEM). * Denotes statistical significance at a p-value < 0.05 determined using a t-test. C) Western blot analysis of whole cell extracts looking at levels of Fas2-GFP and Fas1-HA in WT and *eaf1Δ* yeast. G6PDH is used as a loading control. Representative of n=3.
